# Hepsin-mediated Processing of Uromodulin is Crucial for Salt-sensitivity and Thick Ascending Limb Homeostasis

**DOI:** 10.1038/s41598-019-48300-3

**Published:** 2019-08-23

**Authors:** Eric Olinger, Jennifer Lake, Susan Sheehan, Guglielmo Schiano, Tomoaki Takata, Natsuko Tokonami, Huguette Debaix, Francesco Consolato, Luca Rampoldi, Ron Korstanje, Olivier Devuyst

**Affiliations:** 1 Institute of Physiology, University of Zurich, Zurich, Switzerland; 2 The Jackson Laboratory, Bar Harbor, USA; 3 San Raffaele Scientific Institute, Milan, Italy

**Keywords:** Nephrology, Kidney

## Abstract

Uromodulin is a zona pellucida-type protein essentially produced in the thick ascending limb (TAL) of the mammalian kidney. It is the most abundant protein in normal urine. Defective uromodulin processing is associated with various kidney disorders. The luminal release and subsequent polymerization of uromodulin depend on its cleavage mediated by the serine protease hepsin. The biological relevance of a proper cleavage of uromodulin remains unknown. Here we combined *in vivo* testing on hepsin-deficient mice, *ex vivo* analyses on isolated tubules and *in vitro* studies on TAL cells to demonstrate that hepsin influence on uromodulin processing is an important modulator of salt transport via the sodium cotransporter NKCC2 in the TAL. At baseline, hepsin-deficient mice accumulate uromodulin, along with hyperactivated NKCC2, resulting in a positive sodium balance and a better adaptation to water deprivation. In conditions of high salt intake, defective uromodulin processing predisposes hepsin-deficient mice to a salt-wasting phenotype, with a decreased salt sensitivity. These modifications are associated with intracellular accumulation of uromodulin, endoplasmic reticulum-stress and signs of tubular damage. These studies expand the physiological role of hepsin and uromodulin and highlight the importance of hepsin-mediated processing of uromodulin for kidney tubule homeostasis and salt sensitivity.

## Introduction

Uromodulin, a protein identified by Tamm and Horsfall in 1950, is the most abundant protein excreted in normal human urine. The protein is kidney-specific, essentially produced by the tubular cells lining the thick ascending limb (TAL) and, to a minor extent, the distal convoluted tubule (DCT) of the nephron. Uromodulin contains 640 amino acids, with 7 *N*-glycosylation sites, 4 epidermal growth factor (EGF)-like domains, a zona pellucida (ZP) domain and a C-terminal GPI-anchoring site, is released in urine where it forms high-molecular weight polymers that constitute the matrix of urine casts^1^. Multiple lines of evidence support the biological relevance of uromodulin, which is encoded by the *UMOD* gene. Rare missense mutations in *UMOD* induce aberrant trafficking of mutant uromodulin, leading to endoplasmic reticulum (ER) retention and ER stress that cause autosomal dominant tubulointerstitial kidney disease (ADTKD)^2,3^. On the other hand, genome-wide association studies (GWAS) revealed that common variants in the *UMOD* promoter region, which drive variable expression levels of uromodulin, are linked with increased risks of chronic kidney disease, calcium stones and hypertension in the general population^4–6^.

Multiple physiological roles for uromodulin have been proposed, mostly following studies in knockout (*Umod*^*−/−*^) mice. These roles reflect the structural features of uromodulin and they include: (i) protection against intrarenal and tubular calcium oxalate crystallization (Ca^2+^-binding EGF-like domains)^7^; (ii) defense against urinary tract infections mediated by uropathogenic *E. coli* (high-mannose residues binding to type 1 fimbriae)^8^; and (iii) regulation, in the TAL, of the sodium cotransporter NKCC2 and renal outer medullary potassium channel ROMK by the modulation of their activity and surface abundance, thereby affecting avidity for NaCl and ability to concentrate the urine^9–12^. *UMOD* variants that are associated with hypertension are known to lead to an increase of uromodulin expression and NKCC2 activity in humans^13^. Whether the physiological role of uromodulin is mediated by its cellular processing or apical release into the urine remains unknown.

The luminal release of uromodulin is mediated by a proteolytic cleavage at the C-terminal end of the protein, directly downstream of the ZP domain^14^. ZP domains are essential for the assembly into extracellular polymers and are present in many extracellular eukaryotic proteins including egg coat proteins ZP1-3 and α and β-tectorins^15^. Recently, Brunati *et al*. showed that the transmembrane serine protease hepsin co-localizes with uromodulin in the TAL and is required for the release and subsequent ZP-mediated polymerization of uromodulin. In fact, hepsin cleavage removes an external hydrophobic patch (EHP), located downstream of the ZP domain, which is normally engaged in an inhibitory interaction with an internal hydrophobic patch (IHP)^15,16^. Accordingly, *Hpn* KO mice excrete reduced amounts of uromodulin, that is polymerization-incompetent, establishing uromodulin as a physiological substrate for hepsin^16^. The biological consequences of the lack of hepsin, the fate of misprocessed uromodulin, and the potential repercussions for kidney tubular function remain unknown.

Here we combined studies in the hepsin-deficient *Hpn*^*Hlb320/Hlb320*^ mouse (Hlb320) strain and in isolated TAL segments and derived mouse primary TAL (mTAL) cells, which express endogenous uromodulin and hepsin, to investigate the biological relevance of the hepsin-mediated processing of uromodulin. In control conditions, the functional loss of hepsin leads to cellular accumulation of uromodulin, leading to increased activity of NKCC2 and defective NaCl handling *in vitro* and *in vivo*. In case of high salt exposure, uromodulin excretion dramatically increases in wild-type mice but not in Hlb320 mice, in which uromodulin accumulates inside TAL cells, creating ER stress and tubular dysfunction. These studies expand the physiological role of hepsin and reveal the importance of hepsin-mediated processing of uromodulin for salt handling by the kidney.

## Results

### Characterization of hepsin mutation and consequences on urinary uromodulin

**Figure 1:**
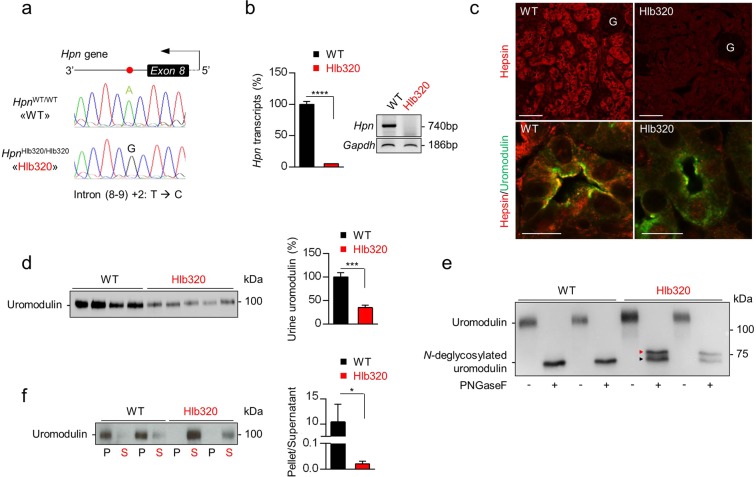
Characterization of hepsin mutation and consequences on urinary uromodulin. (**a**) Antisense strand electropherogram obtained by Sanger sequencing of genomic DNA extracted from *Hpn*^Hlb320/Hlb320^ (Hlb320) and *Hpn*^WT/WT^ (WT) kidney. Above the chromatograph is shown a schematic representation of the corresponding region of *Hpn* gene with the red dot highlighting the transition of A to G in the second base pair in the 3′ splice site of exon 8 (affecting the splicing donor site in intron 8), corresponding to a T to C transition on the sense strand. (**b**) Transcript level of *Hpn*, assessed by RT-qPCR on total kidney extracts from Hlb320 and WT mice using primers directed against exons 8 and 9. A normalization factor based on 6 different housekeeping genes was used. N = 4; bars indicate mean ± s.e.m.; ****p ≤ 0.0001 (Unpaired two-tailed *t* test). *Inset: Hpn* 3′ RT-PCR amplification product (exon 8 to stop codon) from Hlb320 and WT kidneys. Predicted size of amplification product is 740 base pairs. *Gapdh* is used as a loading control. (**c**) *Upper panels:* Immunofluorescence analysis for hepsin (red) in mouse kidney sections from Hlb320 and WT mice. Scale bar: 50 µm. *Lower panels*: High magnification immunofluorescence analysis for hepsin (red) and uromodulin (green) in mouse kidney sections from Hlb320 and WT mice. Scale bar: 10 µm; G, glomerulus. (**d**) Western blot analysis of urinary uromodulin excretion in Hlb320 and WT mice (loading according to urinary creatinine). Densitometric analysis shown next to the blot. Bars indicate mean ± s.e.m.; ***p ≤ 0.001 (Unpaired two-tailed *t* test). (**e**) Representative western blot analysis of urinary uromodulin with or without peptide-*N*-glycosidase F (PNGase F), which removes *N*-linked oligosaccharides from glycoproteins. Red and black arrowheads indicate longer and shorter isoforms, respectively. The equivalent of 0.5 µg and 1 µg of creatinine were loaded for the WT and Hlb320 urine, respectively. (**f**) Representative western blot analysis of urinary uromodulin isolated from the pellet (P) or the supernatant (S) fractions after polymerization assay performed on urine from Hlb320 or WT mice. Densitometric analysis of pellet/supernatant uromodulin indicating uromodulin’s propensity to engage in polymeric structures is shown next to the blot. N = 3; bars indicate mean ± s.e.m.; *p ≤ 0.05 (Unpaired two-tailed *t* test). Uncropped image of blots can be found in Supplementary Figure S8.

The ENU-induced mouse strain Hlb320 harbors a missense mutation in the 3′ splice site of exon 8 of the *Hpn* gene encoding hepsin^17^. The original strain was fully backcrossed to the C57BL/6 J strain in order to eliminate random mutations. The *Hpn*^Hlb320/Hlb320^ mice (hereafter referred to as Hlb320) are born at Mendelian ratios, and are viable and fertile. Genomic DNA analysis confirmed a thymine to cytosine substitution at the second nucleotide position in the 3′ splice site of exon 8 of *Hpn* (Fig. 1a), with only ∼5% residual *Hpn* mRNA expression detected in Hlb320 kidneys by RT-qPCR, no RT-PCR product (exon 8 to stop codon) (Fig. 1b), and no hepsin protein detected in the kidney and TAL apical membrane in Hlb320 mice (Fig. 1c). In contrast to *Hpn*^*−/−*^ mice, the Hlb320 mice used in this study do not display hypothyroidism^17^, which was shown to reduce kidney expression of uromodulin^18^. To substantiate the effects of hepsin deletion, we analyzed the excretion levels of uromodulin and found it strongly decreased in the urine of Hlb320 mice (35.1 ± 5.0% of WT) (Fig. 1d). *N*-deglycosylation using PNGase F shifted the molecular weight of urinary uromodulin from 100 kDa to ~75 kDa in both Hlb320 and WT mice, but revealed two distinct uromodulin isoforms in Hlb320 urine (Fig. 1e, red and black arrowhead), most likely reflecting alternative uromodulin cleavage products^16^. Ultracentrifugation-based polymerization assay^15^, which concentrated uromodulin in the pellet fraction in WT urine, resulted in a complete shift of Hlb320 urine uromodulin in the supernatant fraction - suggesting incapacity of assembling into polymers (Fig. 1f). Collectively, these results show that the loss of hepsin in Hlb320 mice strongly impairs the urinary release of uromodulin, with misprocessing (alternative cleavage) and loss of polymerization.

### Uromodulin accumulation *in vivo* and consequences in the kidney

**Figure 2:**
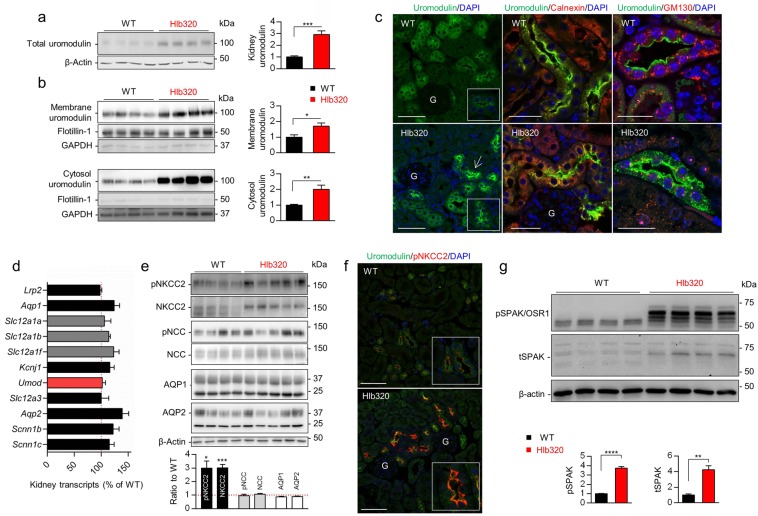
Uromodulin accumulation *in vivo* and consequences in the kidney. (**a**) Western blot analysis of total kidney uromodulin from Hlb320 and WT mice. β-actin is blotted as a loading control and densitometric analysis is shown next to the blot. Bars indicate mean ± s.e.m.; ***p ≤ 0.001 (Unpaired two-tailed *t* test).(**b**) *Upper panels*: Western blot analysis for uromodulin on kidney fractions enriched in large membranes/plasma membrane (“membrane fraction”) from Hlb320 and WT mice. Integral plasma membrane protein flotillin-1 and GAPDH are blotted as positive and negative controls, respectively. Densitometric analysis with normalization to flotillin-1 is shown next to the blot. Bars indicate mean ± s.e.m.; *p ≤ 0.05 (Unpaired two-tailed *t* test). *Lower panels*: Western blot analysis for uromodulin on kidney fractions enriched in cytoplasmic proteins/small vesicles (“cytosolic fraction”) from Hlb320 and WT mice. Cytosolic protein GAPDH and flotillin-1 are blotted as positive and negative controls, respectively. Densitometric analysis with normalization to GAPDH is shown next to the blot. Bars indicate mean ± s.e.m.; **p ≤ 0.01 (Unpaired two-tailed *t* test). (**c**) Representative immunofluorescence analysis for uromodulin (green) and ER chaperone calnexin (red) and Golgi protein GM130 (red) on kidney sections from Hlb320 and WT mice, as indicated. Nuclei are stained in blue with DAPI. Scale bar: 50 µm and 25 µm for co-localization studies; G, glomerulus. (**d**) Transcript levels of major renal transporters and channels as assessed by RT-qPCR on total kidney extracts from Hlb320 mice and WT mice. A normalization factor based on 6 different housekeeping genes was used and results are expressed relative to WT expression (red dotted line). N = 4. Bars indicate mean ± s.e.m. *Lrp2*, megalin; *Aqp1*, aquaporin-1; *Slc12a1a*, NKCC2A; *Slc12a1b*, NKCC2B; *Slc12a1f*, NKCC2F; *Kcnj1*, ROMK; *Umod*, uromodulin; *Slc12a3*, NCC; *Aqp2*, aquaporin-2; *Scnn1b*, β-ENaC; *Scnn1c*, γ-ENaC. (**e**) Western blot analysis in total kidney lysates from Hlb320 and WT mice. A representative β-actin blot is shown as loading control. Densitometric analysis (relative to WT kidneys: red dotted line) is shown below the blots. Bars indicate mean ± s.e.m.; *p ≤ 0.05; ***p ≤ 0.001 (Unpaired two-tailed *t* test). (**f**) Representative immunofluorescence analysis for uromodulin (green) and phosphorylated NKCC2 (at position Thr96, red) in kidney sections from Hlb320 and WT mice. Nuclei are stained in blue with DAPI. Scale bar: 50 µm; G, glomerulus. (**g**) Western blot analysis for phosphorylated SPAK (Ser373)/phosphorylated OSR1 (Ser325) and total SPAK in total kidney lysates from Hlb320 and WT mice. β-actin is blotted as a loading control. Densitometric analysis reports levels normalized to β-actin in Hlb320 kidneys relative to WT kidneys. Bars indicate average ± s.e.m.; **p ≤ 0.01, ****p ≤ 0.0001 (Unpaired two-tailed t test). Uncropped image of blots can be found in Supplementary Figure S8.

In line with reduced urinary levels, the loss of hepsin was reflected by a 3-fold increase of uromodulin in Hlb320 kidneys (Fig. 2a), resulting from accumulation in both the membrane and cytosolic fractions and validated by the appropriate markers flotillin-1 and GAPDH, respectively (Fig. 2b). Immunofluorescence staining revealed a strong apical enhancement for uromodulin in most of the uromodulin-positive tubules in Hlb320 kidneys (Fig. 2c, left panels, inset) with a subset of tubules displaying a broader cytosolic signal (Fig. 2c, left panels, arrow). Co-localization studies revealed that cytosolic uromodulin storage was mainly inside the ER and to a lesser extent inside the Golgi apparatus (Fig. 2c, central and right panels). These results show that defective apical cleavage results in the accumulation of uromodulin along the secretory pathway in Hlb320 kidneys. Messenger RNA profiling revealed no transcriptional changes in major renal transporters and channels as well as unchanged levels of *Umod* transcripts in Hlb320 kidneys (Fig. 2d). No compensatory upregulation of serine proteases prostasin (*Prss8*), mucin 1 (*Muc1*) and dipeptidyl-peptidase IV (*Dpp4*), which are expressed in TAL^16^, were observed (not shown).

The loss of hepsin in Hlb320 kidneys was reflected by a significant upregulation of total and phosphorylated (i.e. active) (Thr96 and Thr101) NKCC2, primarily in the renal cortex, as evidenced by western blotting (Fig. 2e) and immunofluorescence (Fig. 2f & Suppl. Fig. S1, Suppl. Fig. S2a, left panels). These changes were TAL-specific since, in the DCT, the expression of the total and phosphorylated forms of the Na^+^, Cl^−^ cotransporter NCC were not altered in Hlb320 kidneys, despite a high expression level of hepsin in that segment^16^. Water channels expressed in the proximal tubule (AQP1) and collecting duct (AQP2) were also unchanged (Fig. 2e). The increased phosphorylation of NKCC2 is most likely driven by increased levels of activated STE20/SPS1-related proline-alanine–rich kinase (SPAK) and oxidative stress response 1 kinase (OSR1) as pSPAK/pOSR1 levels were markedly higher in NKCC2-positive TAL tubules of Hlb320 mice (Fig. 2g and Suppl. Fig. S2a). In addition, uromodulin accumulation at the apical membrane *per se* might exert a stabilizing role for NKCC2. In line, the apical membrane insertion of NKCC2 is reduced and the signal for pNKCC2 is decreased in *Umod*^*−/−*^ kidneys (Suppl. Fig. S2b). As uromodulin is known to regulate the trafficking of ROMK^10^, we investigated the expression of ROMK in the TAL by performing co-localization studies with uromodulin in Hlb320 and WT mice. As shown by a representative immunofluorescence in Suppl. Fig. S3, we could not detect any alteration in ROMK staining intensity nor pattern in Hlb320 kidneys.

### Altered TAL function and NaCl balance in Hlb320 mice

At baseline, Hlb320 mice showed a similar body weight, water intake, BUN and plasma osmolality levels compared to WT controls (Tables 1 and 2). Hlb320 mice aged 6 months showed a significantly reduced diuresis with hypernatremia and hyperchloremia (Table 2).

**Table 1:** Physiological parameters in 2-month-old Hlb320 and WT mice (Baseline vs. furosemide vs. water deprivation).

	Baseline			Furosemide			Water deprivation		
	WT	Hlb320	n	WT	Hlb320	n	WT	Hlb320	n
Body weight (BW; g)	23.2 ± 0.5	22.6 ± 0.6	5/5						
Daily Water Intake (mL/gBW)	0.17 ± 0.01	0.15 ± 0.01	5/5						
Diuresis (µL/min*gBW)	0.0204 ± 0.0011	0.024 ± 0.002	5/5	0.062 ± 0.013^†^	0.066 ± 0.018	5/5	0.008 ± 0.002 ††	0.005 ± 0.002 ††	5/5
Urinary Na^+^ (nmol/ng creat)	0.19 ± 0.01	0.23 ± 0.01**	5/5	1.18 ± 0.21^††^	3.02 ± 0.6*^,††^	5/5	0.28 ± 0.04	0.23 ± 0.04	5/3^‡^
Urinary Cl^−^ (nmol/ng creat)	0.33 ± 0.01	0.32 ± 0.01	5/5	1.29 ± 0.23^†^	3.25 ± 0.71*^,†^	5/5	0.41 ± 0.06	0.31 ± 0.04	5/3^‡^
Urinary Osmo (mOsm/KgH_2_O)	3914 ± 189	3560 ± 194	5/5	665 ± 103^††††^	500 ± 69^††††^	5/5	6380 ± 906^†^	5865 ± 545	5/3^‡^
Urinary Creatinine (mg/dL)	124 ± 8.1	118 ± 5.4	5/5	15.0 ± 3.9^††††^	7.1 ± 2.5^††††^	5/5	158 ± 10.4^†^	190 ± 17.4^††^	5/3^‡^
Blood urea nitrogen (mg/dL)	23.3 ± 1.0	23.0 ± 1.0	6/5						
Plasma Na^ +^ (mmol/L)	154 ± 0.8	153 ± 0.7	6/5				/	/	
Plasma Cl^−^ (mmol/L)	114 ± 0.8	116 ± 0.7	6/5				131 ± 1.8^†††^	126 ± 0.6*^,†††^	4/5
Plasma Osmo (mOsm/KgH_2_O)	325 ± 1	320 ± 3	6/4				358 ± 6^††^	330 ± 1**	6/4

Values represent means ± s.e.m.; n: number of mice; ^‡^, urine volume insufficient for analyses in 2/5 Hlb320 mice during water deprivation.

Unpaired two-tailed *t* test: *p ≤ 0.05; **p ≤ 0.01; ***p ≤ 0.001, WT vs. Hlb320.

Paired two-tailed *t* test: ^†^p ≤ 0.05; ^††^p ≤ 0.01; ^†††^p ≤ 0.001; ^††††^p ≤ 0.0001, Intervention vs. baseline.

Furosemide, 0.01 mg/gBW in 0.9% saline, IP; Water deprivation during 24 h; WT, C57BL/6J-*Hpn*^*WT/WT*^; Hlb320, C57BL/6J-*Hpn*^Hlb320/Hlb320^.

**Table 2:** Physiological parameters in 6-month-old Hlb320 and WT mice (Control conditions vs. NaCl loading).

	Control			NaCl loading		
	WT	Hlb320	n	WT	Hlb320	n
Body weight (BW; g)	31.9 ± 0.8	30.7 ± 1.1	5/5	32.2 ± 1.2	24.6 ± 1.4**^,$$^	6/5
Daily Water Intake (mL/gBW)	0.15 ± 0.02	0.12 ± 0.01	5/5	0.57 ± 0.18	3.28 ± 0.26****^,$$$$^	6/5
Systolic blood pressure (mmHg)	112.9 ± 2.8	106.4 ± 2.9	6/9	125.7 ± 4.2^††^	101.5 ± 2.5***	6/9
Diuresis (µL/min*gBW)	0.046 ± 0.006	0.021 ± 0.002**	5/5	0.201 ± 0.049^$^	1.632 ± 0.144****^,$$$$^	6/5
Urinary Na^+^ (nmol/ng creat)	0.20 ± 0.02	0.21 ± 0.02	5/5	2.24 ± 0.34^$$$^	27.29 ± 3.79****^,$$$^	6/5
FeNa^+^ (%)	0.10 ± 0.03	0.08 ± 0.01	5/5	1.04 ± 0.34^$^	9.89 ± 1.64***^,$$$^	5/4
Urinary Cl^−^ (nmol/ng creat)	0.39 ± 0.04	0.31 ± 0.03	5/5	2.26 ± 0.36^$$^	26.00 ± 3.48****^,$$$$^	6/5
Urinary Ca^2+^ (nmol/ng creat)	0.0029 ± 0.0008	0.0015 ± 0.0004	5/5	0.0060 ± 0.0012	0.0893 ± 0.0122***^,$$$$^	5/5
Urinary Mg^2+^ (nmol/ng creat)	0.144 ± 0.008	0.129 ± 0.006	5/5	0.118 ± 0.016	0.283 ± 0.031***^,$$^	6/5
Urinary Osmo (mOsm/KgH_2_O)	1524 ± 212	2000 ± 273	5/5	1204 ± 90	747 ± 20***^,$$^	6/5
Urinary Creatinine (mg/dL)	41.0 ± 9.3	53.5 ± 7.4	5/5	18.0 ± 2.3^$^	1.4 ± 0.2***^,$$$^	6/5
Blood urea nitrogen (mg/dL)	25.3 ± 1.3	21.3 ± 0.6*	5/5	21.5 ± 1.8	13.2 ± 2.1*^,$$^	6/5
Creatinine clearance (mL/min*gBW)	0.029 ± 0.007	0.020 ± 0.005	5/5	0.046 ± 0.013	0.032 ± 0.007	4/4
Plasma Na^+^ (mmol/L)	147 ± 0.6	152 ± 0.6***	5/5	151 ± 2.2	182 ± 4.6***^,$$$^	6/4
Plasma Cl^−^ (mmol/L)	109 ± 0.4	113 ± 0.7**	5/5	112 ± 2.2	140 ± 4.2***^,$$$^	6/5
Plasma Ca^2+^ (mmol/L)	2.29 ± 0.02	2.27 ± 0.02	5/5	2.43 ± 0.04^$^	2.05 ± 0.04****^,$$^	6/5
Plasma Mg^2+^ (mg/dL)	2.61 ± 0.08	2.69 ± 0.04	5/5	2.96 ± 0.13	2.29 ± 0.28*	6/5
Plasma Osmo (mOsm/KgH_2_O)	333 ± 3	341 ± 1	5/5	339 ± 1	377 ± 9**^,$$^	6/5

Values represent means ± s.e.m.; n: number of mice.

Unpaired two-tailed *t* test: *p ≤ 0.05; **p ≤ 0.01; ***p ≤ 0.001; ****p ≤ 0.0001, WT vs. Hlb320; ^$$^p ≤ 0.01; ^$$$^p ≤ 0.001; ^$$$$^p ≤ 0.0001, Intervention vs. baseline

Paired two-tailed *t* test: ^††^p ≤ 0.01, Intervention vs. baseline.

NaCl loading, 2% NaCl in drinking water for 2 months; WT, C57BL/6J-*Hpn*^*WT/WT*^; Hlb320, C57BL/6J-*Hpn*^Hlb320/Hlb320^.

**Figure 3:**
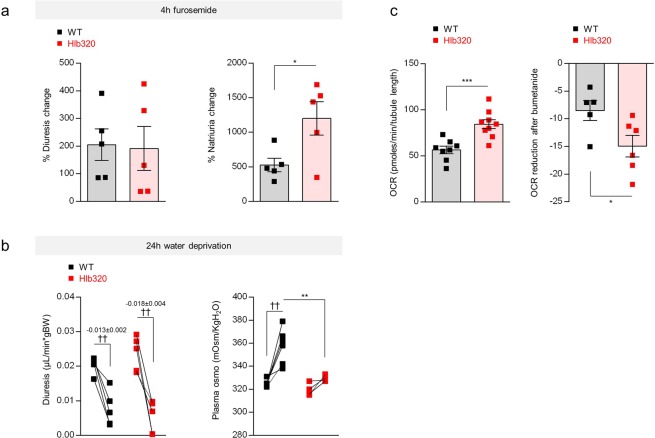
Altered TAL function and NaCl balance in Hlb320 mice. (**a**) *Furosemide test*: Percentage (%) of change in diuresis (left panel, µL/min*gBW) and natriuria (right panel, nmol/ng creat) during 4 hours following an intraperitoneal injection of furosemide (0.01 mg/g of body weight) in Hlb320 mice (red) and WT mice (black). Bars indicate mean ± s.e.m. Each dot represents a mouse; Unpaired two-tailed *t* test: *p ≤ 0.05. (**b**) *Water deprivation test*: Diuresis (µL/min*gBW) and plasma osmolality (mOsm/KgH_2_O) at baseline and during/after a 24-hour period of water deprivation in Hlb320 mice (red) and WT mice (black). Each dot represents a mouse; Unpaired two-tailed *t* test: **p ≤ 0.01; Paired two-tailed *t* test: ^††^p ≤ 0.01. (**c**) Oxygen consumption rate (OCR, pmoles/min/tubule length) in freshly isolated TALs from Hlb320 (red) and WT (grey) mice, as assessed using the Seahorse technology© at baseline (left) and its reduction after bumetanide application (100 µM) (right). Each dot represents a pooled collection of ±70 TALs. Bars indicate mean ± s.e.m.; *p ≤ 0.05; ***p ≤ 0.001 (Unpaired two-tailed *t* test).

Injection of the loop diuretic furosemide, which blocks NKCC2, led to a major, 2-fold increased response in terms of Na^+^ (and Cl^−^) excretion in Hlb320 compared to WT mice (Fig. 3a; Table 1). Water deprivation induced a numerically larger reduction in urine output in Hlb320 vs. WT mice; this increased ability to conserve water completely prevented the rise in plasma osmolality in Hlb320 vs. WT mice (Fig. 3b; Table 1).

We next assessed whether the lack of hepsin affects the metabolism and NKCC2 activity in isolated TAL segments. Freshly isolated TAL tubules from Hlb320 and WT mice displayed a higher rate of oxygen consumption (84.4 ± 4.9 vs. 56.5 ± 4.0 pmoles/min/tubule length, Hlb320 vs. WT, p = 0.0006) (Fig. 3c) and a more pronounced drop of oxygen consumption rate after application of the loop diuretic bumetanide (−14.9 ± 2.0 vs. −8.5 ± 1.8 pmoles/min/tubule length, Hlb320 vs. WT, p = 0.042) (Fig. 3c).

These data demonstrate that the lack of hepsin leads to NKCC2 hyperactivation in the TAL of Hlb320 kidneys, with major alterations in salt and water handling *in vivo*.

Because of the protective effect of uromodulin against uropathogenic *E. coli*^8^, we also tested the susceptibility of Hlb320 mice to bladder colonization by transurethral inoculation of type-1 fimbriated uropathogenic *E. coli*. We could not detect any differences in bladder bacterial titers compared to WT mice (5.2 × 10^6^ ± 4.8 × 10^5^ CFU/gBladder vs. 4.4 × 10^6^ ± 1.2 × 10^6^ CFU/gBladder, N = 7–9, p = 0.46) (Suppl. Fig. S4).

### Altered uromodulin processing and NKCC2 activation in Hlb320 mTAL cells

**Figure 4:**
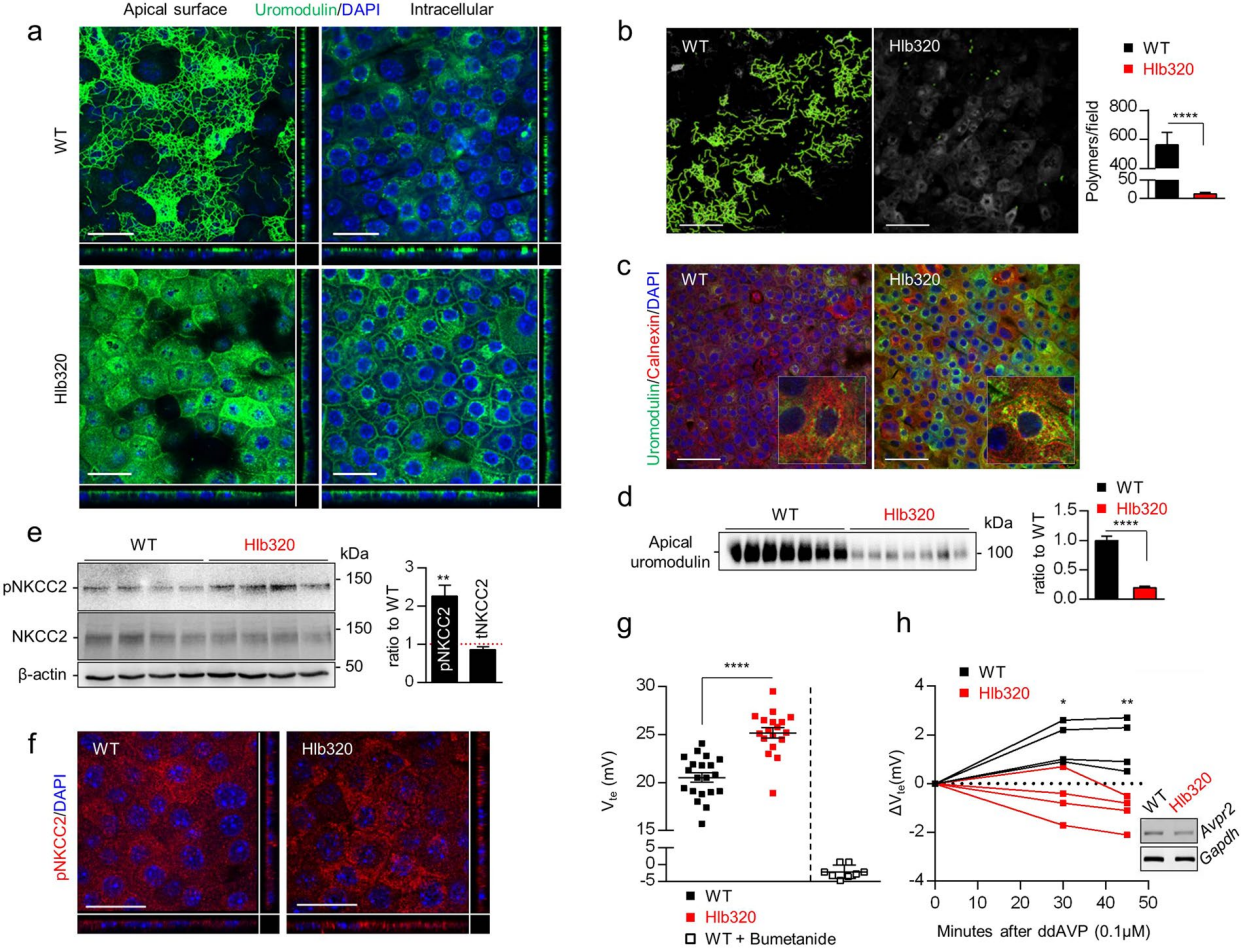
Altered uromodulin processing and NKCC2 activation in Hlb320 mTAL cells. (**a**) Representative immunofluorescence analysis for uromodulin (green) in permeabilized mTAL cells derived from Hlb320 and WT mice. A view of the apical cell surface and through the nuclear plan (intracellular), with the z-plan reconstructions, are shown. Nuclei are stained in blue with DAPI. Scale bar: 25 µm. (**b**) Representative output picture and result of polymer number per field quantification using ImageJ Ridge Detection in mTAL cells from Hlb320 and WT mice. N = 15 fields from 3 mTAL cultures. Scale bar: 50 µm; bars indicate mean ± s.e.m.; ****p ≤ 0.0001 (Unpaired two-tailed *t* test). (**c**) Representative immunofluorescence analysis for uromodulin (green) and ER chaperone calnexin (red) in permeabilized mTAL cells derived from Hlb320 and WT mice. *Inset*: zoom on single cell. Nuclei are stained in blue with DAPI. Scale bar: 50 µm. (**d**) Representative western blot analysis of uromodulin secreted in the apical compartment of mTAL cells derived from Hlb320 and WT mice. Same apical volumes were loaded in each lane. Densitometric analysis is shown next to the blot. Bars indicate mean ± s.e.m.; ****p ≤ 0.0001 (Unpaired two-tailed *t* test). (**e**) Western blot analysis for phosphorylated (Thr96 and Thr101) and total NKCC2 in mTAL cells from Hlb320 and WT mice. β-actin is shown as a loading control. Each lane represents a different mTAL cell culture. Densitometric analysis (relative to WT) is shown next to the blots. Bars indicate mean ± s.e.m.; **p ≤ 0.01 (Unpaired two-tailed *t* test). (**f**) Representative immunofluorescence analysis for phosphorylated NKCC2 (at position Thr96, red) in permeabilized mTAL cells derived from Hlb320 and WT mice. Z-plan reconstructions are shown on the picture borders. Nuclei are stained in blue with DAPI. Scale bar: 25 µm. (**g**) Transepithelial voltage (V_te_, mV) recordings using a chopstick electrode on mTAL cells derived from Hlb320 (red), WT (black) mice and WT cultures after application of apical bumetanide (100 µM; white). Each dot represents a filter of primary TAL cells. Bars indicate mean ± s.e.m.; ****p ≤ 0.0001 (Unpaired two-tailed *t* test). (**h**) Evolution of transepithelial voltage (V_te_, mV) after basolateral ddAVP application (0.1 µM) in mTAL cells derived from Hlb320 (red) or WT (black) mice. Each dot represents a mTAL cell culture. Bars indicate mean ± s.e.m.; *p ≤ 0.05; **p ≤ 0.01 (Unpaired two-tailed *t* test). The RT-PCR amplification product for *Avpr2* is shown for Hlb320 and WT mTAL cultures. *Gapdh* is shown as a loading control. Uncropped image of blots can be found in Supplementary Figure S8.

We next used the mTAL primary cell system, with conserved processing and secretion of endogenous uromodulin^19^ to investigate the cell-autonomous consequences of hepsin deletion. Compared to mTAL cells derived from WT kidneys, mTAL cells from Hlb320 kidneys completely lacked uromodulin polymers on the apical surface; displayed apical membrane and cytosol/ER accumulation of uromodulin; and released considerably less uromodulin in the apical compartment (Fig. 4a–d). Reduced secretion of polymerization-incompetent uromodulin could also be shown in WT mTAL cells transduced with shRNA directed against *Hpn* mRNA, leading to knockdown of hepsin (Suppl. Fig. S5). Of note, mTAL cells derived from Hlb320 mice display robust expression levels of *Umod* mRNA (160 ± 24% of WT mTAL cells, p = 0.10, data not shown). Hlb320 mTAL cells displayed increased levels of phosphorylated NKCC2 by western blotting (Fig. 4e) and by immunofluorescence (Fig. 4f), translating into a significantly increased bumetanide-inhibitable transepithelial voltage (Fig. 4g). Furthermore, in contrast to WT cells, Hlb320 mTAL cells were unable to increase their transepithelial voltage when treated with basolateral ddAVP, a potent stimulator of NKCC2 phosphorylation and apical membrane insertion^20^, suggesting maximal recruitment of NKCC2 at baseline in these cells (Fig. 4h). Taken together, these studies in primary mTAL cells confirm all the changes observed in the Hlb320 mice and substantiate a cell-autonomous interplay between hepsin-mediated uromodulin processing and regulation of NKCC2 activity.

### NaCl loading drives uromodulin accumulation and tubular damage in Hlb320 mice

**Figure 5:**
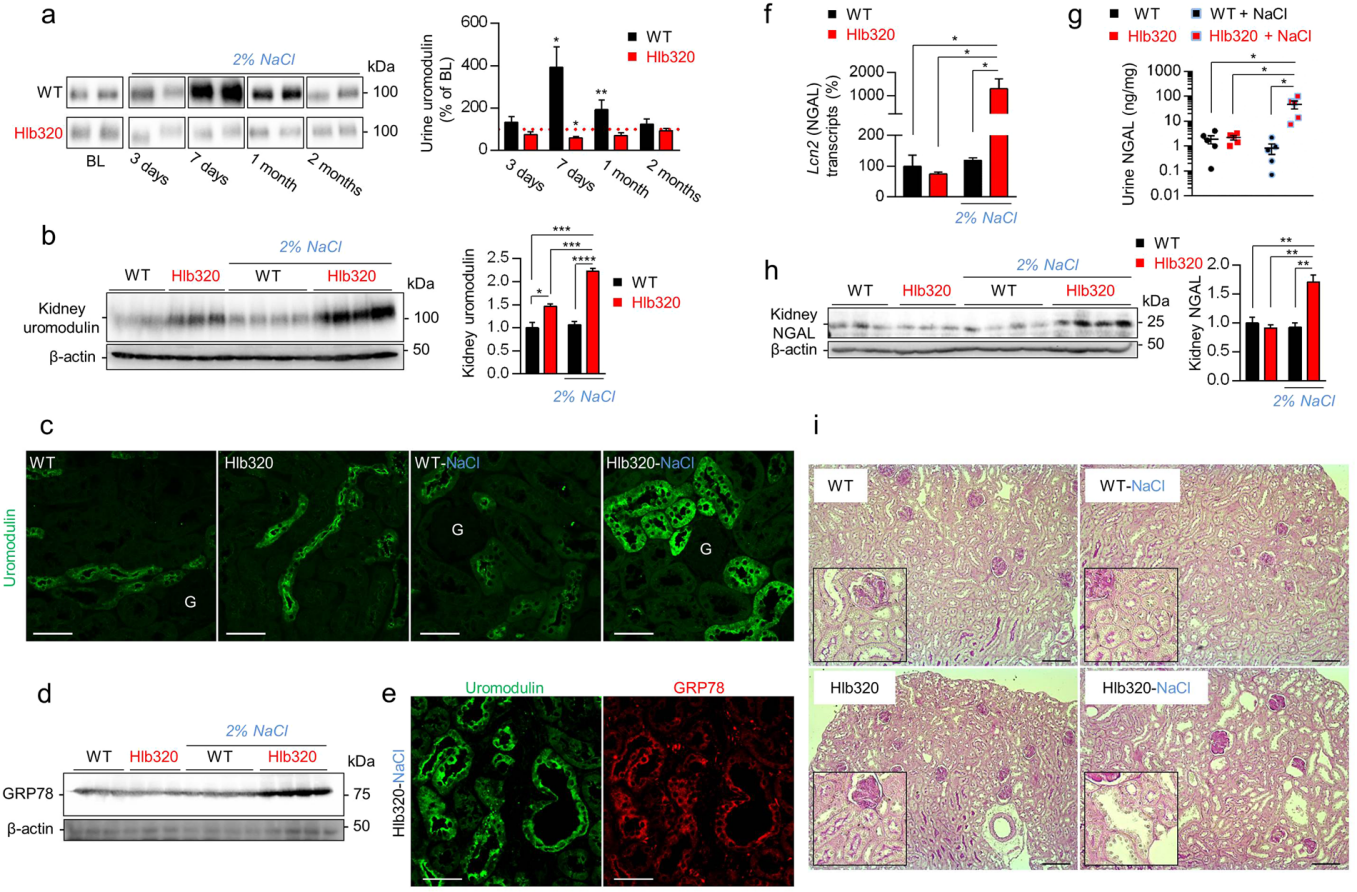
NaCl loading drives uromodulin accumulation and tubular damage in Hlb320 mice. (**a**) Representative western blot analysis for urinary uromodulin excreted in Hlb320 and WT mice at baseline (BL) and at different time points of an oral 2% NaCl loading protocol. Western blot loading is normalized to urinary creatinine to account for urine dilution and cropped images are shown for ease of representation. The densitometric analysis indicates relative changes of urinary uromodulin levels at indicated time points as compared to the baseline values for that same mouse for samples that have been loaded on the same gel. N = at least 3. Bars indicate mean ± s.e.m.; *p ≤ 0.05; **p ≤ 0.01 (Paired two-tailed *t* test, values at indicated time point vs. baseline values). (**b**) Western blot analysis for kidney uromodulin levels in Hlb320 and WT mice under control conditions and after 2 months of NaCl loading. β-actin is shown as a loading control. Densitometric analysis relative to control WT. Bars indicate mean ± s.e.m.; *p ≤ 0.05; ***p ≤ 0.001; ****p ≤ 0.0001 (Unpaired two-tailed *t* test). (**c**) Representative immunofluorescent analysis for uromodulin (green) on kidney sections from Hlb320 and WT mice at control conditions and after 2 months of NaCl loading, as indicated. Scale bar: 50 µm; G, glomerulus. (**d**) Western blot analysis of kidney GRP78 levels in Hlb320 and WT mice under control conditions and after 2 months of NaCl loading. β-actin is shown as a loading control. (**e**) Representative immunofluorescent analysis for uromodulin (green) and ER-stress marker GRP78 (red) on kidney sections from NaCl loaded Hlb320 mice. Scale bar: 50 µm. (**f**) Transcript levels of *Lcn2* (NGAL), assessed by RT-qPCR on total kidney extracts from Hlb320 and WT mice at control conditions and after 2 months of NaCl loading, as indicated. A normalization factor based on 6 different housekeeping genes was used and levels are expressed relative to control WT. N = at least 4; bars indicate mean ± s.e.m.; *p ≤ 0.05 (Unpaired two-tailed *t* test). (**g**) ELISA for NGAL (ng/mg creatinine, log 10 scale) in the urine of Hlb320 and WT mice under control conditions and after 2 months of NaCl loading, as indicated. Each dot represents urine collected from a different mouse. Bars indicate mean ± s.e.m.; *p ≤ 0.05 (Unpaired two-tailed *t* test). (**h**) Western blot analysis of kidney NGAL levels in Hlb320 and WT mice under control conditions and after 2 months of NaCl loading, as indicated. β-actin is shown as a loading control. Densitometric analysis relative to control WT. Bars indicate mean ± s.e.m.; **p ≤ 0.01 (Unpaired two-tailed *t* test). (**i**) Representative histological analysis (Periodic acid-Schiff) of the renal cortex of Hlb320 and WT mice at control conditions and after 2 months of NaCl loading, as indicated. Scale bar: 100 µm. Uncropped image of blots can be found in Supplementary Figure S8.

In view of the established link between dietary NaCl and expression of uromodulin^21^, we challenged WT and Hlb320 mice with a NaCl loading protocol (2% NaCl in drinking water) for 2 months. In contrast to WT mice, which showed a marked increase in levels of urinary uromodulin starting at day 7 and lasting 1 month, Hlb320 mice showed no increase (and even a decrease at day 7) in the urinary levels of uromodulin after NaCl loading (Fig. 5a). As a consequence of this defective urinary release, the NaCl loading led to a massive further increase of uromodulin accumulation in the kidneys of Hlb320 mice after 2 months, which was not observed in WT mice (Fig. 5b). These dramatic changes in uromodulin accumulation induced by NaCl loading were confirmed in the TAL profiles from Hlb320 vs. WT mice (Fig. 5c). Because of the ER-accumulation of uromodulin in Hlb320 mice, we investigated the levels of ER chaperone and ER stress marker GRP78 and detected an upregulation in NaCl loaded Hlb320 kidneys (Fig. 5d), emanating from uromodulin-positive TAL tubules (Fig. 5e). Neutrophil gelatinase-associated lipocalin (NGAL) is a biomarker of renal tubule injury that is selectively expressed after ER stress and is causally linked to progression of renal disease^22,23^. The transcript levels of NGAL (encoded by *Lcn2*) were strongly upregulated in NaCl loaded Hlb320 vs. WT kidneys (Fig. 5f) with a parallel increase in urinary (Fig. 5g) and kidney (Fig. 5h) levels of NGAL. Histological analysis of PAS-stained kidney sections reveals distal tubular damage with massively dilated tubule profiles in salt-loaded Hlb320 mice, but not in Hlb320 mice under control conditions or in NaCl loaded WT mice (Fig. 5i).

### Salt-losing (Bartter-like) phenotype in NaCl loaded Hlb320 mice

**Figure 6:**
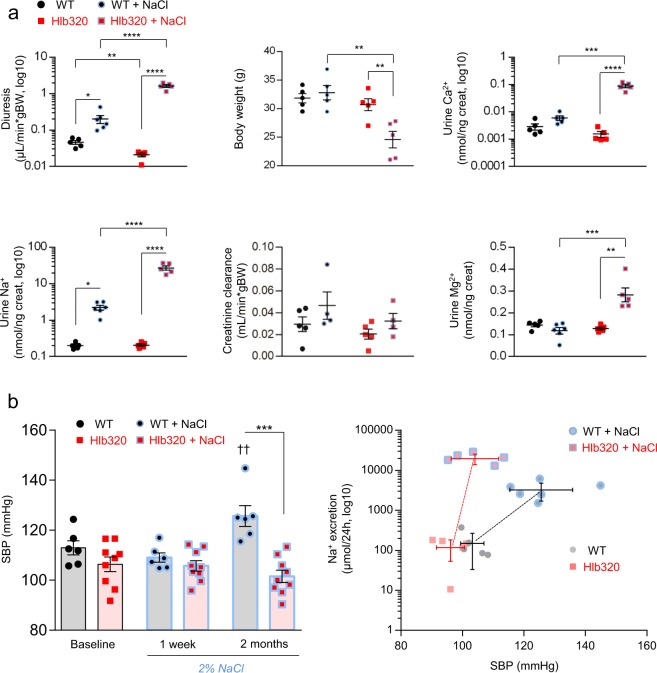
Bartter-like phenotype in NaCl loaded Hlb320 mice. (**a**) Evolution of physiological parameters related to kidney function in Hlb320 (red) and WT (black) mice in control conditions and after NaCl loading, as indicated. Each dot represents a mouse. Bars indicate mean ± s.e.m.; *p ≤ 0.05; **p ≤ 0.01; ***p ≤ 0.001; ****p ≤ 0.0001 (Unpaired two-tailed *t* test). (**b**) *Left panel*: Systolic blood pressure (SBP) averages as assessed by tail cuff measurements on the same mice during 2 months. Each single dot represents an average of 3–5 consecutive daily SBP recordings in Hlb320 (red) and WT (black) mice at control conditions and at different reporting periods after NaCl loading, as indicated. Bars indicate mean ± s.e.m.; ***p ≤ 0.001 (Unpaired two-tailed *t* test); ^††^p ≤ 0.01 (Paired two-tailed *t* test: SBP at 2 months of NaCl loading versus baseline). *Right panel*: Pressure-natriuresis relationship obtained by plotting Na^+^ excretion (µmol/24 h) against SBP for Hlb320 (red) and WT (black) mice at control conditions and after NaCl loading, as indicated. Error bars indicate means ± s.d.

After 2 months of NaCl loading, Hlb320 mice developed a severe renal phenotype that resembles Bartter syndrome, including massive polyuria with NaCl, Ca^2+^ and Mg^2+^ wasting, decline in body weight with a concomitant rise in plasma osmolality, hypocalcemia and hypomagnesemia. Glomerular filtration was not deteriorated as shown by creatinine clearance and BUN levels (reduced BUN reflecting most probably muscle wasting) (Fig. 6a & Table 2) and plasma potassium levels were unchanged (4.1 ± 0.1 mmol/L in Hlb320 vs. 4.2 ± 0.1 mmol/L in WT mice, n = 4, after 1 month of NaCl loading). The link between ER stress and TAL dysfunction was supported by experiments on mTAL cells using tunicamycin, a potent inducer of ER stress by blocking *N*-linked glycosylation. Tunicamycin treatment increased NGAL expression, reduced the expression of total NKCC2 (and to a lesser extend of phosphorylated NKCC2) and led to a drop in the transepithelial voltage and the vectorial Ca^2+^ transport in mTAL cells (Suppl. Fig. S6).

The changes in tubular function were reflected by differences in blood pressure as assessed by tail cuff recordings over the period of 2 months. In accordance with previous reports^11^, WT mice increased their systolic blood pressure (SBP) over the course of the 2% NaCl loading. In sharp contrast, Hlb320 mice showed no increase in SBP after 1 and 2 months of NaCl loading, most likely reflecting the observed NaCl wasting and the dysfunction of the TAL segment (Fig. 6b, left panel & Suppl. Fig. S7). These alterations in renal NaCl avidity were reflected by a shift in the relationship between Na^+^ intakes with SBP, when comparing Hlb320 vs. WT mice (Fig. 6b, right panel). In a subset of mice, we analyzed the phosphorylation ratio of NKCC2 after 1 month of 2% NaCl loading, since the difference in SBP response became apparent at that timepoint. A defective phosphorylation level of NKCC2 with an inverted phospho/total NKCC2 ratio was detected specifically in the NaCl loaded Hlb320 mice (data not shown). Collectively, these results indicate that the hepsin-dependent processing of uromodulin is critical to regulate the NaCl-handling capacity of the TAL segment exposed to high salt intake. In such conditions, defective hepsin leads to a major accumulation of uromodulin causing ER stress and NGAL expression, with TAL dysfunction and abnormal regulation of NaCl handling and blood pressure.

## Discussion

Our studies show, for the first time, that the hepsin-mediated release and polymerization of uromodulin modulates the salt transporting activity of TAL cells *in vivo* and *in vitro*. The intracellular accumulation of uromodulin in hepsin-deficient mice and cells drives the hyperactivation of the cotransporter NKCC2, reflected by significant modifications of the response to pharmacological and physiological testing. Exposure to NaCl triggered hepsin-mediated urinary uromodulin release in control mice, contrasting with intracellular accumulation of uromodulin causing epithelial dysfunction and defective blood pressure regulation in hepsin-deficient mice. Collectively these data show that the processing of uromodulin in the kidney tubule is crucial for salt handling and blood pressure regulation.

We provide the first report of a kidney phenotype linked to the functional loss of the serine protease hepsin. Mice lacking hepsin display increased levels of functionally active (phosphorylated) NKCC2 at baseline, with a positive NaCl balance and an increased ability to conserve water during testing. In light of previous studies in uromodulin-deficient and -overexpressing mice, NKCC2 hyperactivation in Hlb320 mice is most likely driven by the misprocessing and cellular accumulation of uromodulin^9,13^. In fact, Hlb320 and *Umod*^*−/−*^ mice present a mirror phenotype with respect to water handling, a feature that could reflect the reduced function of NKCC2 in the former and the hyperactivation of NKCC2 in the latter. The observed phenotype of the Hlb320 mice is biologically meaningful: NKCC2 is selectively active in the TAL segment that expresses uromodulin and it is a crucial player in blood pressure control as demonstrated by the effects of genetic (Type 1 Bartter syndrome) and pharmacological (loop diuretics) inhibition^24^.

NKCC2 is regulated at the level of its trafficking and phosphorylation, with an expanding number of molecular mechanisms involved. These include the highly homologous SPAK and OSR1 kinases, which themselves are under the control of upstream WNK kinases^25^. The levels of uromodulin expression have been shown to correlate directly with the activity of NKCC2, most likely through regulation of intracellular chloride levels and modulation of the chloride sensitive WNK-SPAK/OSR1 pathway^9,13^. Activity of this pathway is increased in the kidneys of Hlb320 mice, along with an increased phosphorylation of NKCC2. The mechanism by which uromodulin modulates SPAK/OSR1 activity could involve potentialization of with-no-lysine kinase (WNK) activity, as previously suggested^9^. The facts that (i) soluble uromodulin (truncated at the GPI-anchoring site) is unable to activate NKCC2^13^; (ii) NKCC2 and uromodulin are distributed in close spatial vicinity^9^; and (iii) uromodulin and NKCC2 are sharing lipid raft localization^26^ raise the possibility that uromodulin acts as a scaffold for WNK-SPAK/OSR1-dependent activation of NKCC2. The data presented here extend the role of uromodulin in NKCC2 activation, by showing that the dynamics of uromodulin processing modulates NKCC2 activity. Of note, the polymerization of uromodulin is not required for the NKCC2 regulatory effect. The fact that total NKCC2 levels are increased in Hlb320 kidneys suggests an additional effect of the lipid raft-associated uromodulin on the turnover of NKCC2, similar to what has been shown for MAL/VIP17^27^.

The complete absence of endogenous uromodulin polymers in primary TAL cells derived from hepsin-deficient mice confirm the requirement of this protease for uromodulin polymerization. The physiological relevance of uromodulin polymerization and matrix formation is still unclear^1^. Under specific ionic conditions, uromodulin polymers form a water-impermeable matrix, which could be important to ensure the water impermeability of the TAL segment^28^. However, Hlb320 mice completely lack urinary uromodulin polymers and still they display an increased capacity of water retention. The fact that complete absence of uromodulin in *Umod* KO mice leads to a urinary concentrating defect^29^, that is not encountered in Hlb320 mice, might indicate that small quantities of monomeric uromodulin in the lumen are sufficient to assure water impermeability. Alternatively, the activation of NKCC2 in the TAL, resulting from the accumulation of uromodulin in Hlb320 mice, may increase the medullary osmotic gradient and, in turn, drive a higher urinary concentrating ability. The reasonable amounts of glycosylated uromodulin still secreted by the Hlb320 mice may also explain their intact protection against uropathogenic *E. coli*^30^. Contrary to 6 month-old Hlb320 mice, which show a positive NaCl balance, young Hlb320 mice display a trend for Na^+^ wasting in urine. This observation suggests that the phenotype develops with aging and may reflect a progressive accumulation of uromodulin in TAL cells. Because the uromodulin processing machinery is conserved in primary mTAL cells, monolayers derived from WT and Hlb320 mice represent a valuable tool for future mechanistically investigations regarding the impact of GPI-anchored uromodulin on cellular homeostasis.

Several lines of evidence point towards an interplay between uromodulin, salt, and blood pressure. Trudu *et al*.^13^ showed that hypertensive individuals homozygous for an *UMOD* promoter variant driving higher uromodulin expression levels display an exaggerated response to furosemide when compared to individuals harboring the *UMOD* genotype associated with lower levels of uromodulin expression. This observation strongly suggests that *UMOD* promoter variants are contributing to the regulation of NKCC2 activity and NaCl transport, via their influence on uromodulin expression. In rats, high dietary NaCl intake increased uromodulin mRNA and protein levels in kidneys^21^. In humans, urinary uromodulin levels positively correlated with NaCl intake^6,31^. Furthermore, subjects with the highest uromodulin levels after high NaCl diet were also more prone to develop salt-sensitive hypertension, suggesting a higher salt-sensitivity of the TAL^31^. Based on these observations, we tested the effect of NaCl exposure on the hepsin-mediated processing of uromodulin. Our experiments reveal that the transient increase in uromodulin excretion induced by NaCl exposure, observed in control mice, is solely mediated by hepsin - without alternative cellular pathways to excrete uromodulin or to reduce its production. In turn, the defective processing of uromodulin in the Hlb320 mice leads to its accumulation in the ER, leading to ER stress (GRP78) and renal tubular injury (NGAL, histology). These changes are paralleled by a severe tubular dysfunction: after 2 months of NaCl loading, Hlb320 mice developed NaCl wasting and a loss of NaCl-sensitivity evidenced by a shift in the relationship between Na^+^ intakes with systolic blood pressure. Hepsin-mediated processing of uromodulin is thus critical to sustain the NaCl-handling capacity of the TAL and, more generally, the role of the kidney tubule in blood pressure regulation.

It should be pointed that NaCl loading caused uncompensated polyuria with weight loss and severe hypernatremia in the Hlb320 mice. The increase in plasma sodium levels was reminiscent of that observed in *Avpr2*^Y/−^ pups with nephrogenic diabetes insipidus or in *Slc12a1*^*−/−*^ pups with Bartter syndrome type 1^32,33^. Of note, the Bartter-like phenotype with uncompensated polyuria and hypernatremia was not observed after 1 month of NaCl loading in Hlb320 mice (except for reduced systolic blood pressure), suggesting a time-dependent mechanism compatible with progressive ER accumulation of uromodulin.

Finally, these studies in hepsin-deficient systems emphasize the vulnerability of the TAL, a kidney tubule segment that is constantly producing, at high level, a complex protein requiring a challenging ER quality control, while being involved in highly regulated transport processes involved in NaCl and divalent cation handling. It should be noted that deficient cleavage of uromodulin, which is supposed to take place at the apical membrane^16^, leads not only to the expected uromodulin accumulation at the apical membrane but also to an engorgement of the secretory pathway, and particularly the ER, with the protein. Due to its complex tertiary structure (24 disulfide bridges), uromodulin is challenging in terms of ER proteostasis as reflected by a particularly long ER residency^34^. The delicate balance between ER proteostasis and ER stress is illustrated here, with robust increase in GRP78 and NGAL markers and dysfunction of TAL cells of the Hlb320 mice exposed to NaCl load, mimicking what is observed in the ER-storage disease caused by mutated uromodulin^3^.

## Materials and Methods

### Mouse lines

Hlb320 mice were generated at The Jackson Laboratory as part of an ENU mutagenesis program as previously described^17^. The Hlb320 strain was backcrossed for >10 generations to the C57BL/6J strain before the experiments. The nucleotide missense change was confirmed by Sanger sequencing using an Applied Biosystems 3730 DNA Analyzer (Applied Biosystems, Foster City, CA). All animals were housed in temperature- and humidity-controlled pathogen-free facility with free access to acidified water and a standard rodent chow diet (5kK2 LabDiet, Brentwood, MO). Animal procedures were carried out at The Jackson Laboratory, Bar Harbor, ME and at the University of Zurich, Zurich, Switzerland according to protocols approved by The Jackson Laboratory’s Animal Care and Use Committee and the Swiss Cantonal Veterinary Authority (103/2014), respectively.

### Treatment protocols, plasma and urine collection, biochemical analysis

Furosemide test and water deprivation were performed on 8 weeks-old male Hlb320 and WT mice. Furosemide testing was performed by administering a single intraperitoneal injection of furosemide (0.01 mg/g of body weight in 0.9% saline; Sigma-Aldrich, Saint-Louis, MO) with subsequent urine collection during 4 hours. Mice were deprived of water for 24 h, urine was collected during this period and mice were sacrificed thereafter. 16 weeks-old male Hlb320 and WT mice were evaluated at baseline and then administered 2% NaCl in drinking water for 4 or 8 weeks, and sacrificed at the end of the NaCl loading period. Urine was collected using individual metabolic cages (UNO Roestvastaal BV, Zevenaar, The Netherlands) after appropriate training and in a light- and temperature-controlled room. Blood was sampled in the sublingual artery or by cardiac puncture at the time of sacrifice. Blood was centrifuged at 2000 g for 15 minutes at 4 °C in heparin-coated tubes (Sarstedt AG, Nürnbrecht, Germany) in order to separate plasma from cells. Electrolytes, creatinine and urea were measured in urine and/or plasma samples using a Synchron DXC800 analyzer (Beckman Coulter, Fullerton, CA). Plasma and urine osmolality was determined on a multi-sample osmometer (Advanced Instruments Model 2020, Norwood, MA). Urine NGAL levels were measured using an enzyme-linked immunosorbent assay according to the manufacturer’s instructions (EMLCN2, Thermo Fischer Scientific, Waltham, MA). All tests were performed in The Jackson Laboratory, Bar Harbor, ME or the University of Zurich, Zurich, Switzerland according to protocols approved by The Jackson Laboratory’s Animal Care and Use Committee and the Swiss Cantonal Veterinary Authority (103/2014), respectively.

### Isolation, growth and treatment of mouse thick ascending limb cells

Thick ascending limbs from 6- to 8-weeks old male C57BL/6J-*Hpn*^Hlb320/Hlb320^ or C57BL/6J-*Hpn*^*WT/WT*^ mice were isolated and cultured as reported previously^19^. Thick ascending limbs were manually collected under a light microscope and seeded on fibronectin-coated (Corning Inc., Corning, NY) 96-well plastic plates (Thermo Fischer Scientific) containing a culture medium based on DMEM:F12^19^ (Thermo Fischer Scientific) and placed in a humidified chamber at 37 °C and 5% CO_2_. When cellular outgrow reached near confluence, cells were passed using 0.05% Trypsin-EDTA (Thermo Fischer Scientific) to fibronectin and collagen-coated 0.33 cm^2^ PTFE filter membranes (Corning Inc., Corning, NY). After a confluent monolayer of cells was reached, supplemented FBS (Thermo Fischer Scientific) was reduced from 2% to 0.1% (v/v) to allow maximal differentiation. Cells were treated with 100 µM bumetanide (Sigma-Aldrich) to the apical side, with 0.1 µM ddAVP (Sigma-Aldrich) to the basolateral side or with 2.5 µg/mL tunicamycin (Sigma-Aldrich) to the apical and basolateral side. Transepithelial voltage measurements were performed using chopstick electrodes (STX2, World precision instruments, Sarasota, FL) according to manufacturer’s instructions.

### Protein extraction, sample processing and immunoblotting

Mice were euthanized with a lethal dose of isoflurane (Minrad International Inc., Orchard Park, NY) and both kidneys were removed and decapsulated. One kidney was rapidly frozen in liquid nitrogen and grinded using a pestle and mortar. The other kidney was processed for immunohistochemistry and mRNA analysis. Proteins were extracted from grinded tissue or mTAL cells using ice-cold RIPA buffer (Sigma-Aldrich, St.-Louis, MO) with the addition of protease and phosphatase inhibitors (Roche, Basel, Switzerland), followed by a centrifugation for 15 min at 1000 g and 4 °C to remove debris and a brief sonication of the supernatant. When required, proteins were extracted in a sucrose buffer (1 mM EDTA, 20 mM Imidazole and 250 mM Sucrose, pH 7.2) and pellets enriched in large plasma membranes (“membrane fraction”) (17,000 g, 1 h, 4 °C) and in cytoplasmic proteins and cytoplasmic vesicles (“cytosolic fraction”) (200,000 g, 2 h, 4 °C) were sequentially collected. Proteins from urine samples or from apical cell medium were *N*-deglycosylated using PNGaseF (New England Biolabs, Ipswich, MA) according to manufacturer’s instructions. Polymerization assay on urinary samples was performed as previously described. In brief, urine samples were centrifuged at 150,000 g for 3 h at 20 °C. The supernatants were removed and the pellets solubilized in SDS sample buffer for downstream analysis^15^. Protein concentration was assessed using the bicinchoninic acid (BCA) protein assay kit (Thermo Fischer Scientific) and urine sample loading was normalized to creatinine content. Urine samples, cell medium and tissue or cell lysates were mixed with Laemmli sample buffer (Bio Rad Laboratories, Hercules, CA), proteins were separated on 7.5–12% SDS-PAGE gel in non-reducing (uromodulin) or in reducing (all other analyses) conditions and transferred onto PVDF membrane (Bio Rad). Western blotting was performed using established protocols^19^. Densitometric quantification was done with the ImageJ software^35^.

### Histological analysis and immunostaining

Half kidneys were fixed in 4% formaldehyde (Sigma-Aldrich) and embedded in paraffin. Paraffin blocks were cut into 5-µm-thick sections, deparaffinated in xylene and re-hydrated in serial ethanol concentrations. Periodic acid-Schiff staining was performed according to standard protocols and images were captured with a light microscope. For immunofluorescence analysis, epitope retrieval was performed in a 10 mM citric acid solution (pH 6.0) at 98 °C for 10 min in a tissue processor (Milestone Inc., Shelton, CT). Sections were then blocked in PBS containing 3% BSA (Sigma-Aldrich), 30 mM glycine (Sigma-Aldrich), 50 mM NH_4_Cl (VWR international, Radnor, PA) and 0.05% Tween-20 (Merck-Millipore) for 1 hour at room temperature. The samples were incubated with the primary antibody for 2 hours in a humidified chamber at RT or overnight at 4 °C and subsequently with the appropriate AlexaFluor-labeled secondary antibody (1:300, Life Technologies, Carlsbad, CA) for 1 hour at RT. Where appropriate, a second primary antibody and its secondary antibody were applied. TAL monolayers on PTFE filters were fixed for 10 minutes in 2% formaldehyde (Sigma-Aldrich), permeabilized for 30 minutes using 0.5% saponin (Sigma-Aldrich) and blocked overnight at 4 °C in 3% BSA (Sigma-Aldrich). The immune-staining procedures were then similar to the kidney sections. After the last washing step, filters were cut onto a glass slide. Kidney sections and filters were mounted in Prolong Gold Anti-fade reagent containing DAPI (Invitrogen Corp., Waltham, MA) and viewed under a confocal microscope (Leica Microsystems GmbH, Wetzlar, Germany) using a ×63 1.4 NA oil immersion objective. Acquisition parameters were kept identical for the same antibody in different kidney sections. Image processing, quantification of uromodulin polymers/field, relative polymer size and % of field area covered by polymers is performed using ImageJ software and ImageJ Ridge detection^35^.

### Antibodies

The following antibodies were used: sheep anti-uromodulin (K90071C, Meridien Life Science Inc., Cincinnati, OH; 1:500 for WB and 1:300 for IF), sheep-anti uromodulin (ab9029, Abcam; 1:500 for WB), rabbit anti-hepsin (100022, Cayman Chemical, Ann Arbor, MI; 1:50 for IF), mouse anti-flotillin-1 (BD 610821, BD Biosciences, Franklin Lakes, NJ, 1:500 for WB), rabbit anti-GAPDH (2118, Cell Signaling Technology, Danvers, MA; 1:500 for WB), mouse anti-βactin (A5441, Sigma-Aldrich; 1:10 000 for WB), rabbit anti-calnexin (C4731, Sigma-Aldrich; 1:300 for IF), rabbit anti-phospho NKCC2 (R5, phosphorylated Thr96 and Thr101)(gift from Prof. Biff Forbusch, Yale; 1:500 for WB), rabbit anti-phospho NKCC2 (phosphorylated Thr96) (gift from Prof. Jan Loffing, Zurich; 1:300 for IF), rabbit anti-NKCC2 (AB3562P, Merck-Millipore, 1:500 for WB and 1:100 for IF), rabbit anti-phospho NCC (phosphorylated Thr53)(gift from Prof. Jan Loffing; 1:5000 for WB), rabbit anti-NCC (AB3553, Merck-Millipore, 1:500 for WB), rabbit anti-aquaporin 1 (AB2219, Merck Millipore; 1:500 for WB), rabbit anti-aquaporin 2 (A7310, Sigma-Aldrich; 1:1000 for WB), anti-phospho SPAK (Ser373)/phospho OSR1 (Ser325)(07–2273, Merck Millipore; 1:500 for WB and 1:300 for IF), rabbit anti-SPAK (07–2271, Merck Millipore; 1:500 for WB), mouse anti-GM130 (ab169276, Abcam, Cambridge, UK; 1:300 for IF), rabbit anti-NGAL (ab63929, Abcam; 1:500 for WB), rabbit anti-GRP78 (ab21685, Abcam; 1:500 for WB and 1:300 for IF).

### Determination of oxygen consumption rate

Oxygen consumption rate (OCR) was measured in freshly microdissected TAL tubules using the XFp Extracellular Flux Analyzer and the XFp Cell Mito Stress Test kit (Seahorse Bioscience, North Billerica, MA). TAL microdissection was performed as described above with following modifications: TAL’s were collected in XF-Base Medium supplemented with 2 mM L-glutamine, 1 mM sodium pyruvate and 10 mM glucose (pH 7.4) and seeded in the Seahorse XFp Cell Culture Miniplate containing the same medium and kept for 1 h at 37 °C in ambient air. OCR was measured at basal condition and following the injection of bumetanide (Sigma-Aldrich) at a final concentration of 100 µM. After the measurement, tubules were fixed in 4% PFA and tubule length was assessed using ImageJ software^35^.

### Tail cuff manometry

Systolic blood pressure (SBP) was assessed by a non-invasive computerized tail cuff method (Visitech Systems, Apex, NC) as described^36^. Animals were trained for 5 days before the start of the study. SBP was measured following a fixed daily schedule. Multiple consecutive recordings (at least 10) were performed for each animal and then averaged in order to get a SBP of the day. Daily SBP averages were only considered for the final analysis if standard deviations of the individual recordings were below 10 mmHg. SBP were measured this way during at least 3 consecutive days, measurements were then again averaged for each mouse for the reporting period (reporting periods last between 3 and 5 days). SBP reporting periods included a baseline period as well as reporting periods starting 1 week, 1 month and 2 months after the 2% oral NaCl loading protocol started.

### Induction of urinary tract infections

Transurethral instillation of uropathogenic *E. coli* was performed as described^37^. In brief, human UPEC strain J96 (ATCC 700336) was statically grown in LB broth at 37 °C with serial passages to ensure type-1 pili expression. Bacteria were resuspended in PBS and their viable concentration was determined by titration on LB agar plates. 1.65 × 10^8^ CFU in 50 µL were transurethrally instilled in the bladders of 10 week-old female Hlb320 and WT mice. Animals were euthanized 24 h after UPEC instillation, bladders were aseptically removed, homogenized and remaining CFU were determined by LB agar microtiter dilution. The importance of bacterial pili for bladder colonization was verified using a strain of FimH-deficient *E. coli* (W3110) (not shown, kindly provided by Prof. Glockshuber, ETH).

### RNA extraction, reverse transcription, PCR, quantitative PCR

Analysis of gene expression levels in mouse kidneys and TAL cells were performed based on the MIQUE guidelines^38^. In brief, the extraction of total RNA from TAL cells was performed using RNAqueous-Micro kit (Ambion, Huntingdon, UK). Total RNA was extracted from kidney using Aurum^TM^ Total RNA Fatty and Fibrous Tissue Kit (Bio Rad, Hercules, CA), following the protocol of the manufacturer. Contamination by genomic DNA was eliminated by DNAse I treatment. Reverse transcriptase reaction with iScript^TM^ cDNA Synthesis Kit (Bio Rad) was executed with up to1 µg of RNA. When needed, PCR products were size-fractionated on 1.5% agarose gel and stained with EZ-Vision^R^ One (AMRESCO, Solon, OH). The variations in mRNA levels of the target genes were established by relative RT-qPCR with a CFX96^TM^ Real-Time PCR Detection System (Bio Rad) and the iQ^TM^ SYBR Green Supermix (Bio Rad) for the detection of single PCR product accumulation. 100 nM of sense and anti-sense primers were used in a final volume of 20 µl in iQ^TM^ SYBR Green Supermix (Bio Rad) to perform RT-qPCR analyses (in duplicate). Primers specific to targets were designed with Primer3^39^ (Suppl. Table S1). PCR conditions were: 95 °C, 3 min followed by 40 cycles of 15 sec, 95 °C and 30 seconds at 60 °C. The efficiency of each set of primers was determined by dilution curves (Suppl. Table S1). For TAL cell expression studies, *Gapdh* was used as a reference gene. In order to characterize the expression stability of the candidate reference genes in kidney, the program geNorm version 3.4 was applied. The most stable genes are stepwise selected from the investigated gene panel and a common Normalization Factor (NF) can be calculated for the genes selected for the normalization procedure. We selected the 6 tested reference genes to calculate the Normalization Factor^40^.

### Silencing of hepsin expression

Hepsin shRNAs (Santa Cruz Biotechnologies, Dallas, TX) is a pool of concentrated, transduction-ready lentiviral particles containing 3 target-specific constructs that encode 19–25 nt (plus hairpin) shRNA designed to knock down *Hpn* gene expression. The transduction was performed at a concentration of 3.3 × 10^5^ and 6.6 × 10^5^ pfu/ml. Briefly, primary mouse TAL cells were seeded onto collagen-coated filters (Corning Inc., Corning, NY) and lentivirus infections were performed 24 h after plating at around 70% of confluence with 5 µg/ml of polybrene. Cells were incubated for 12 h at 37 °C with culture medium containing the virus, then changed with 0.1% FBS culture medium for other 5 days. Control cells were transduced in parallel with a control shRNA Lentiviral Particles-A (Santa Cruz Biotechnologies).

### Sanger sequencing

Sequencing of the PCR products was done with the BigDye terminator kit (Perkin Elmer, Waltham, MA). A dye-terminator removal process was performed with the multiScreen SEQ_384_. Filter Plate (Merck-Millipore, Billerica, MA) and Sephadex G-50 DNA Grade Fine (Amersham, Buckinghamshire, UK) to purify the sequences reactions which were next analysed with an ABI3100 capillary sequencer (Perkin Elmer).

### Statistical analysis

Values are expressed as arithmetic mean ± standard error of the mean (s.e.m.). Statistical comparisons were performed using an unpaired two-tailed Student’s *t* test (GraphPad Software, La Jolla CA) or a paired two-tailed Student’s *t* test (GraphPad Software, La Jolla CA). *P* values ≤ 0.05 were considered as statistically significant.

## Supplementary information

Supplementary Material

## Data Availability

The datasets generated during and/or analyzed during the current study are available from the corresponding author on reasonable request.
