# Supplementary material for: Hepsin-mediated Processing of Uromodulin is Crucial for Salt-sensitivity and Thick Ascending Limb Homeostasis
Source: Sci Rep. 2019 Aug 23;9:12287. doi: 10.1038/s41598-019-48300-3 (PMC6707305; doi:10.1038/s41598-019-48300-3)
Supplement: Supplementary file 1 — Supplementary Material [file 41598_2019_48300_MOESM1_ESM.pdf]

Hepsin-mediated Processing of Uromodulin is Crucial for Salt-sensitivity and Thick Ascending Limb Homeostasis

Eric Olinger, Jennifer Lake, Susan Sheehan, Guglielmo Schiano, Tomoaki Takata, Natsuko Tokonami, Huguette Debaix, Francesco Consolato, Luca Rampoldi, Ron Korstanje and Olivier Devuyst

<u>Supplementary Material</u>

Supplementary Figures S1 to S8

Supplementary Table S1

Suppl. Fig. S1: Total NKCC2 and phosphorylated NKCC2 in the kidney medulla of Hlb320 mice.

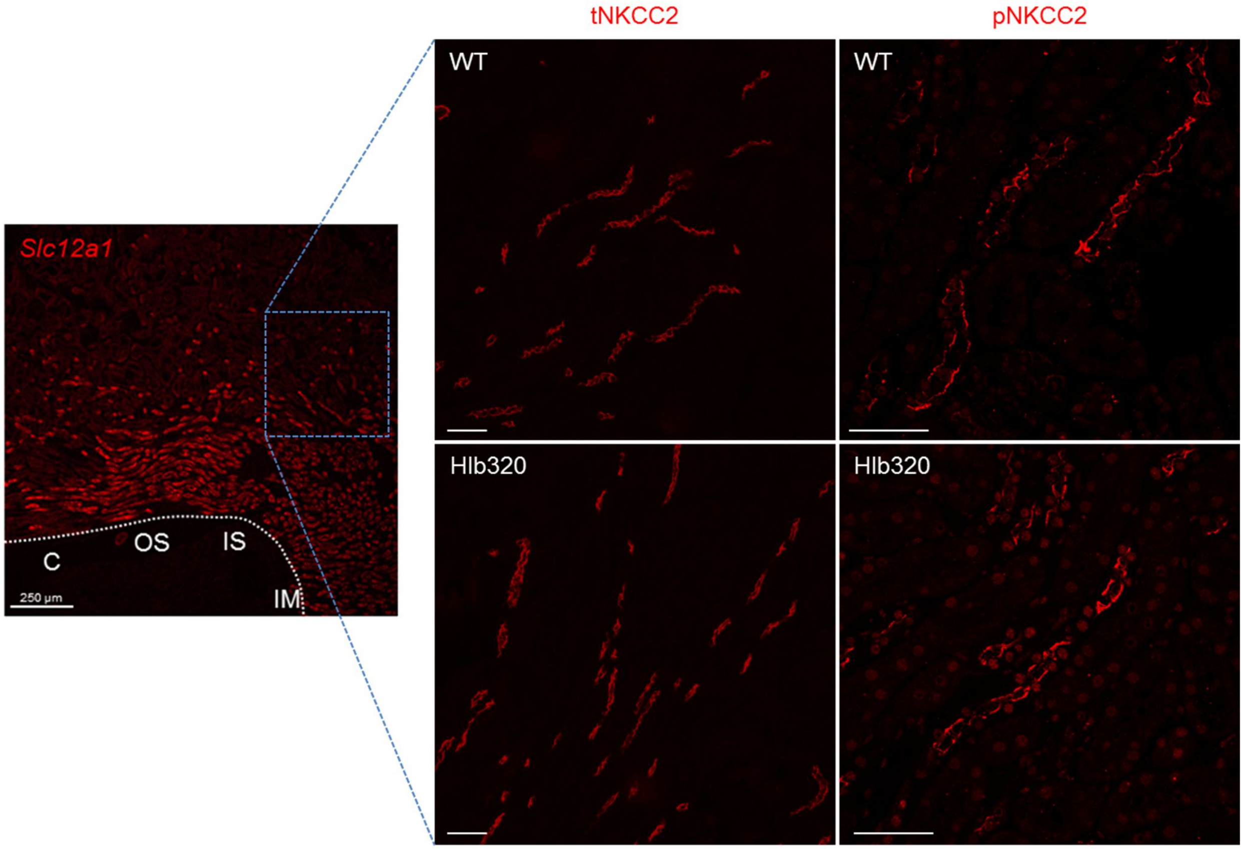

Left panel: NKCC2 expression in the WT murine kidney as assessed by fluorescent *in situ* hybridization for *Slc12a1* (red).
Right panels: Representative immunofluorescence analysis for total NKCC2 and phosphorylated NKCC2 (Thr96) in the inner stripe of outer medulla (dashed blue outline) of kidney sections from Hlb320 and WT mice.
Scale bar right panels: 50µm. C, cortex; OS, outer stripe of the outer medulla; IS, inner stripe of the outer medulla; IM, inner medulla.

Suppl. Fig. S2: SPAK in Hlb320 kidneys and NKCC2 levels in *Umod*$^{-/-}$ mice.

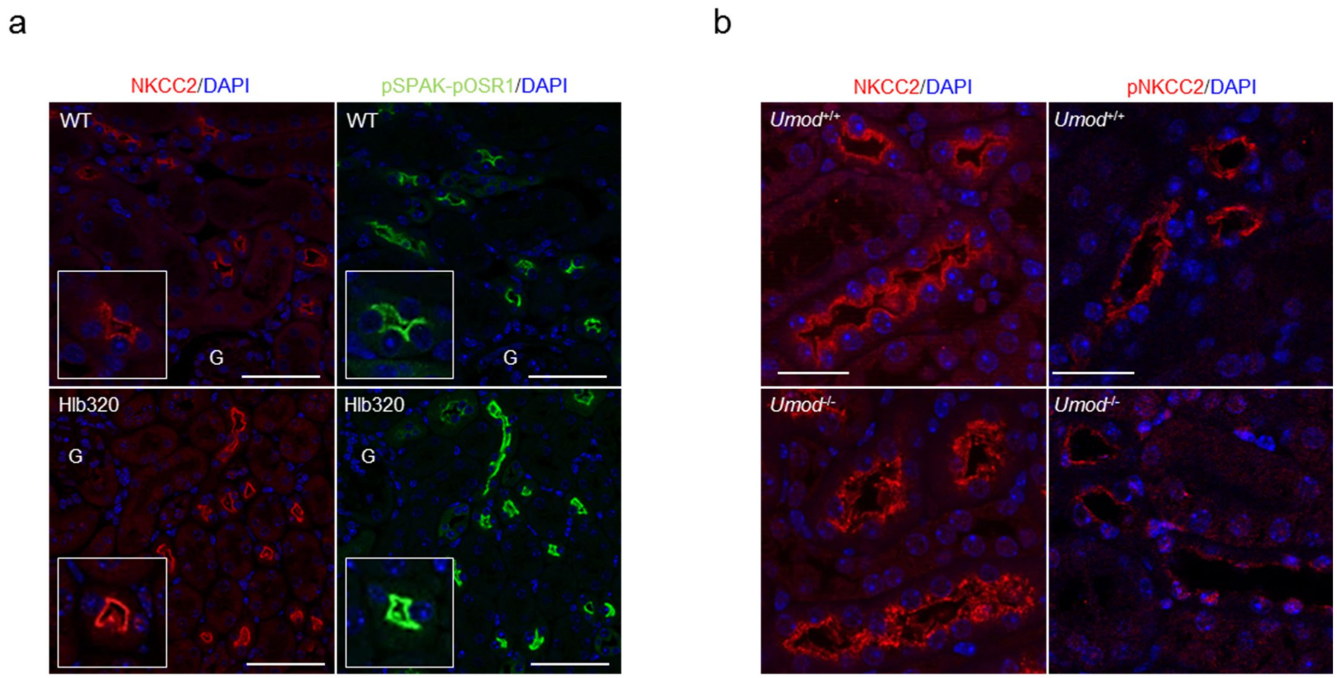

A. Representative immunofluorescence analysis for NKCC2 (red) and phosphorylated SPAK (Ser373)/phosphorylated OSR1 (Ser325) (green) in serial kidney sections from Hlb320 and WT mice. Nuclei are stained in blue with DAPI. Scale bar: 50µm; G, glomerulus.

B. Representative immunofluorescence analysis for total NKCC2 (left panels) and phosphorylated NKCC2 (Thr96, right panels) in kidney sections from *Umod*$^{-/-}$ and WT mice. Nuclei are stained in blue with DAPI. Scale bar: 25µm.

Suppl. Fig. S3: ROMK levels in the kidney cortex of Hlb320 mice.

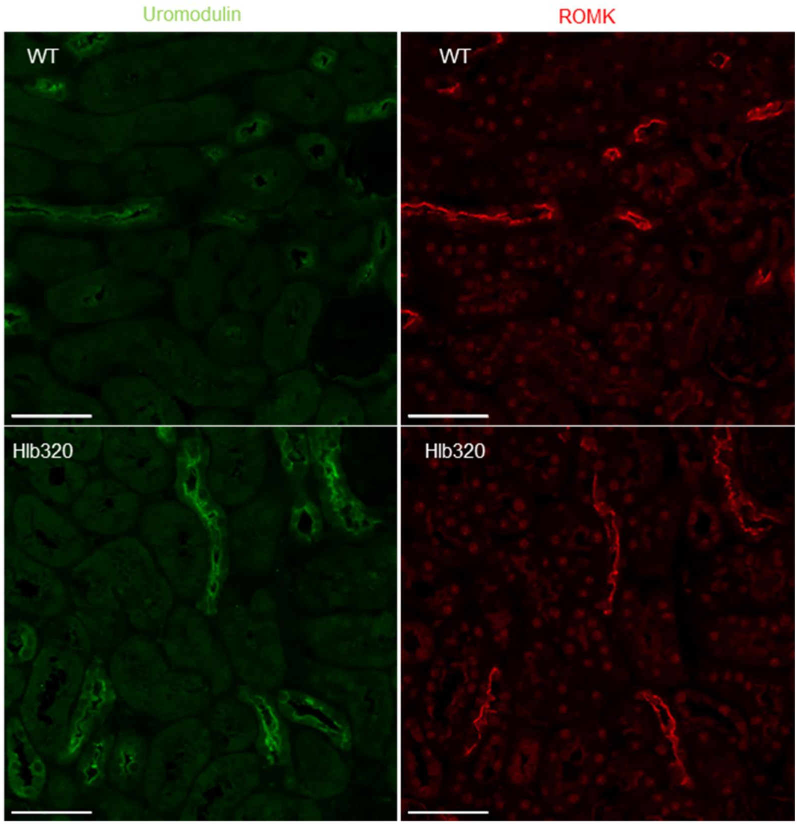

Representative immunofluorescence analysis for Uromodulin (green) and ROMK (red) in serial kidney sections from Hlb320 and WT mice. Scale bar: 50μm.

Suppl. Fig. S4: Bladder colonization by uropathogenic *E.coli* in Hlb320 and WT mice.

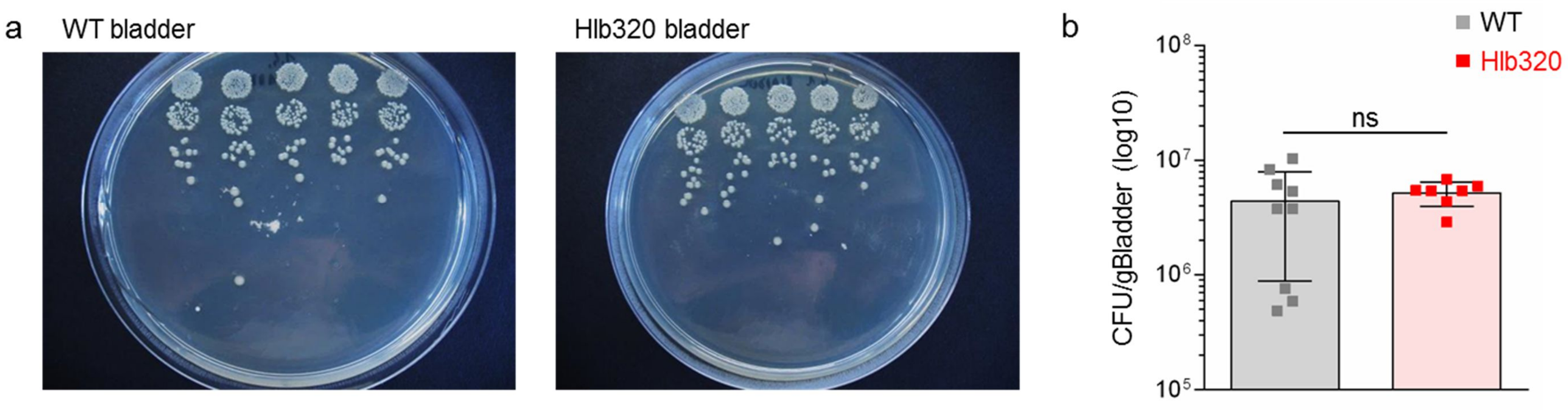

A. Representative pictures of colony-forming units (CFU) on LB agar microtiter dilution plates (10^0 to 10^{-7}, from top to bottom) from Hlb320 and WT bladder homogenates 24h after transurethral inoculation with $1.65x10^8$ CFU of uropathogenic E.*coli* strain J96.

B. Viable bacterial titers in Hlb320 and WT bladders 24h after transurethral inoculation with $1.65x10^8$ CFU of E.*coli* strain J96, as assessed by LB agar microtiter dilutions. Each dot represents a mouse. Bars indicate average ± s.e.m.

Suppl. Fig. S5: Defective polymerization and uromodulin secretion after *Hpn* knockdown in primary TAL cells.

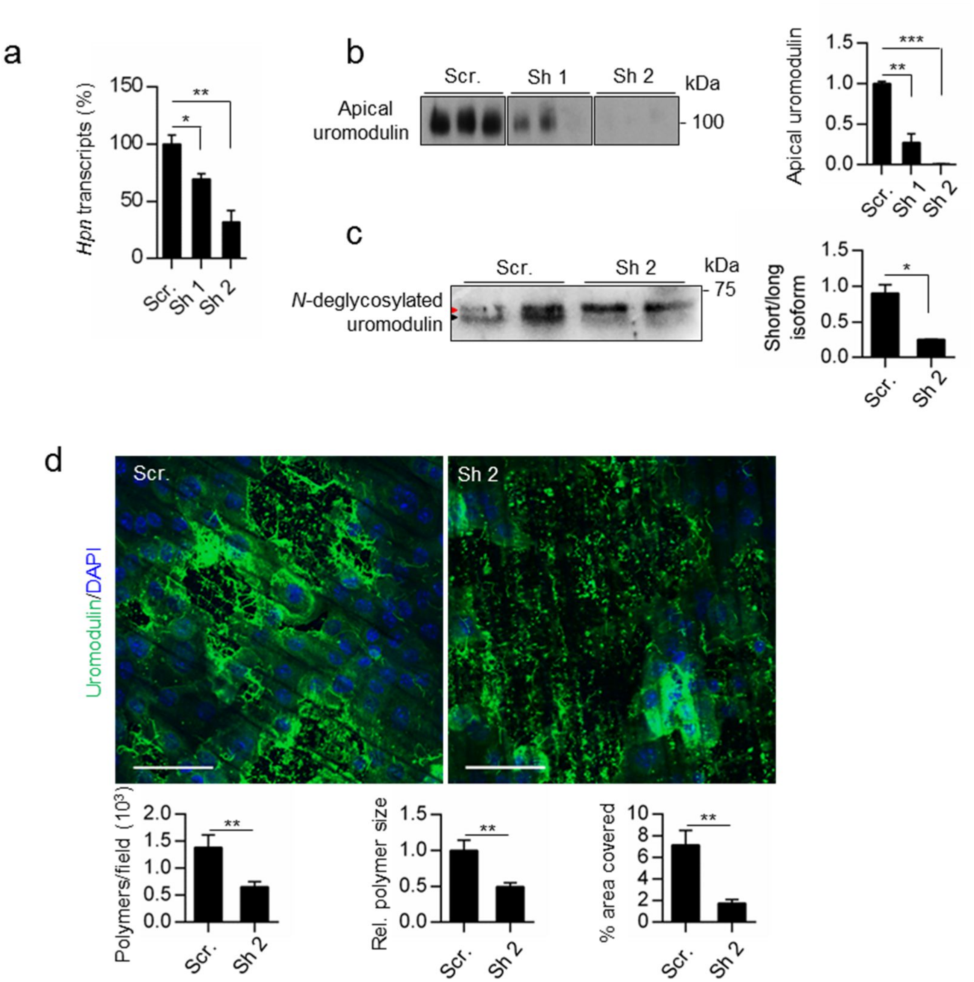

Suppl. Fig. S5: Defective polymerization and uromodulin secretion after *Hpn* knockdown in primary TAL cells.

A. Transcript levels of *Hpn* as assessed by RT-qPCR in WT mTAL cells after lentiviral mediated transduction of either scrambled shRNA (scramble) or shRNA directed against *Hpn* mRNA at two different viral doses (Sh 1: 3.3 x 10^5 and Sh 2: 6.6 x 10^5 pfu/ml), as indicated. Normalization was performed using *Gapdh* expression and results are indicated relative to scramble. N=5 scramble and N=3 for each dose; bars indicate average ± s.e.m.; * $p \leq 0.05$, ** $p \leq 0.01$ (Unpaired two-tailed *t* test).
B. Western blot analysis for uromodulin secreted by mTAL cells into the apical compartment 3 days after transduction with scramble or *Hpn*-targeting shRNAs, as indicated. Equal apical volume was loaded into each lane and on the same gel. Densitometric analysis is performed relative to scramble. Bars indicate average ± s.e.m.; ** $p \leq 0.01$, *** $p \leq 0.001$ (Unpaired two-tailed *t* test). Uncropped image of blots can be found in Supplementary figure S8.
C. Western blot analysis for *N*-deglycosylated uromodulin secreted into the apical compartment of mTAL cells either transduced with scramble or *Hpn*-targeting shRNA. Distinct short and long isoforms are marked with a black and red arrow, respectively. Densitometric analysis of the ratio short/long isoform shows that transduction with hepsin shRNA leads to the specific reduction of the shorter, physiological isoform in this experiment[1]. * $p \leq 0.05$ (Unpaired two-tailed *t* test).
D. Representative immunofluorescence analysis for uromodulin in mTAL cells either transduced with scramble or *Hpn*-targeting shRNA. Nuclei are stained in blue with DAPI. Scale bar: 50µm. Quantification of uromodulin polymers/field, relative polymer size and % of field area covered by polymers is performed using ImageJ software. N=9 fields from 3 mTAL cultures. Bars indicate average ± s.e.m.; ** $p \leq 0.01$ (Unpaired two-tailed *t* test).

Suppl. Fig. S6: ER stress-mediated induction of NGAL and epithelial dysfunction in mTAL cells.

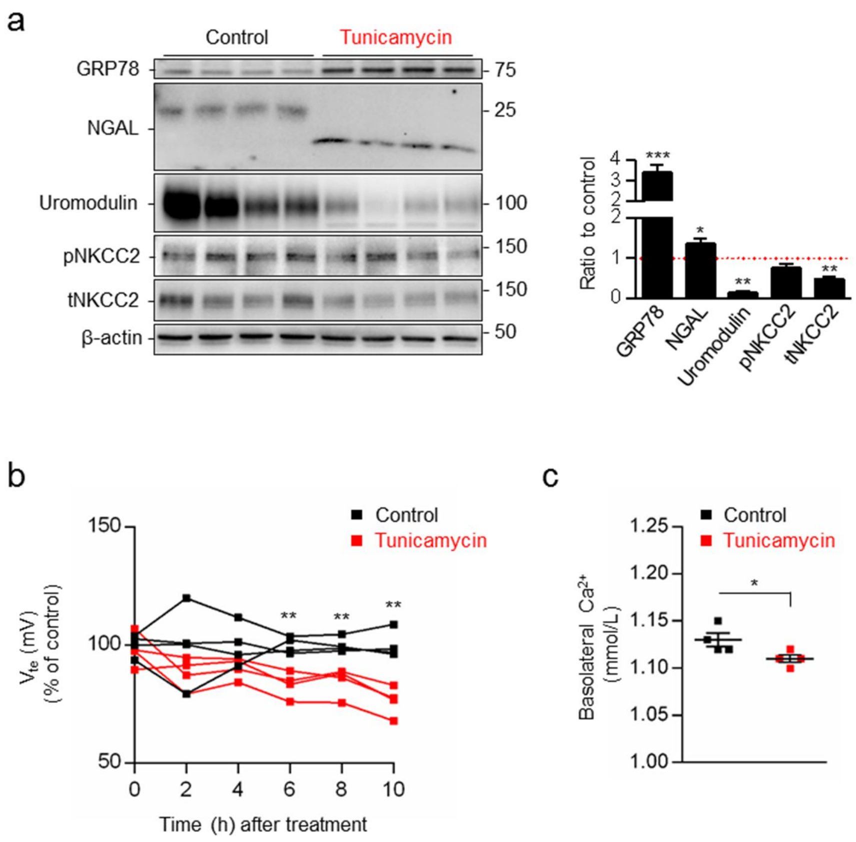

Suppl. Fig. S6: ER stress-mediated induction of NGAL and epithelial dysfunction in mTAL cells.

A. Western blot analysis for GRP78, NGAL, uromodulin, phosphorylated and total NKCC2 in control (0.05% DMSO) and tunicamycin-treated (2.5µg/mL) WT mTAL cells after 10h of treatment (apical and basolateral). The shift of NGAL molecular weight is due to impaired *N*-glycosylation. A representative β-actin is shown as a loading control. Each lane represents a different mTAL cell culture. Densitometric analysis (relative to control) is shown next to the blots. Bars indicate average ± s.e.m.; * $p \leq 0.05$; ** $p \leq 0.01$; *** $p \leq 0.001$ (Unpaired two-tailed *t* test).
B. Evolution of transepithelial voltage (V_{te}, mV) after tunicamycin application (2.5µg/mL) in WT mTAL cells, as compared with control cells (0.05% DMSO). Each dot represents a different mTAL cell culture. ** $p \leq 0.01$ (Unpaired two-tailed *t* test).
C. Ca^{2+} concentrations (mmol/L) in the basolateral compartment of control (0.05% DMSO) and tunicamycin-treated (2.5µg/mL) mTAL cells after 10h of respective treatment. Each dot represents a different mTAL cell culture. * $p \leq 0.05$ (Unpaired two-tailed *t* test).

Suppl. Fig. S7: Systolic blood pressure changes after 1 month of NaCl loading.

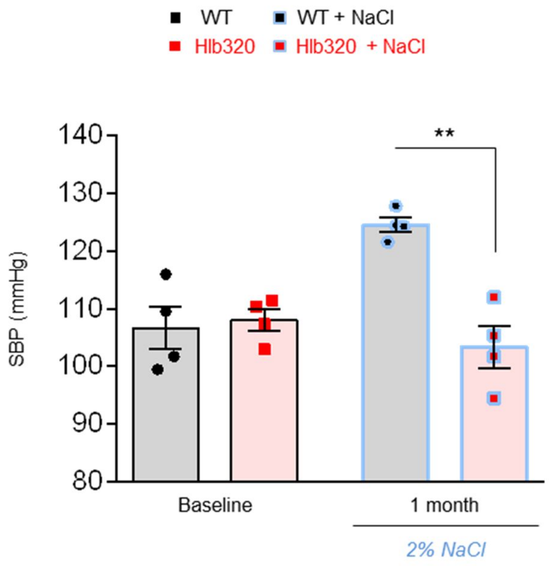

Systolic blood pressure (SBP) averages as assessed by tail cuff measurements in Hlb320 and WT mice under control conditions and after 1 month of NaCl loading, as indicated. Each dot represents a different mouse. Three daily SBP recordings were averaged and reported. Bars indicate average ± s.e.m.; **$p \leq 0.01$ (Unpaired two-tailed *t* test).

Suppl. Fig. S8

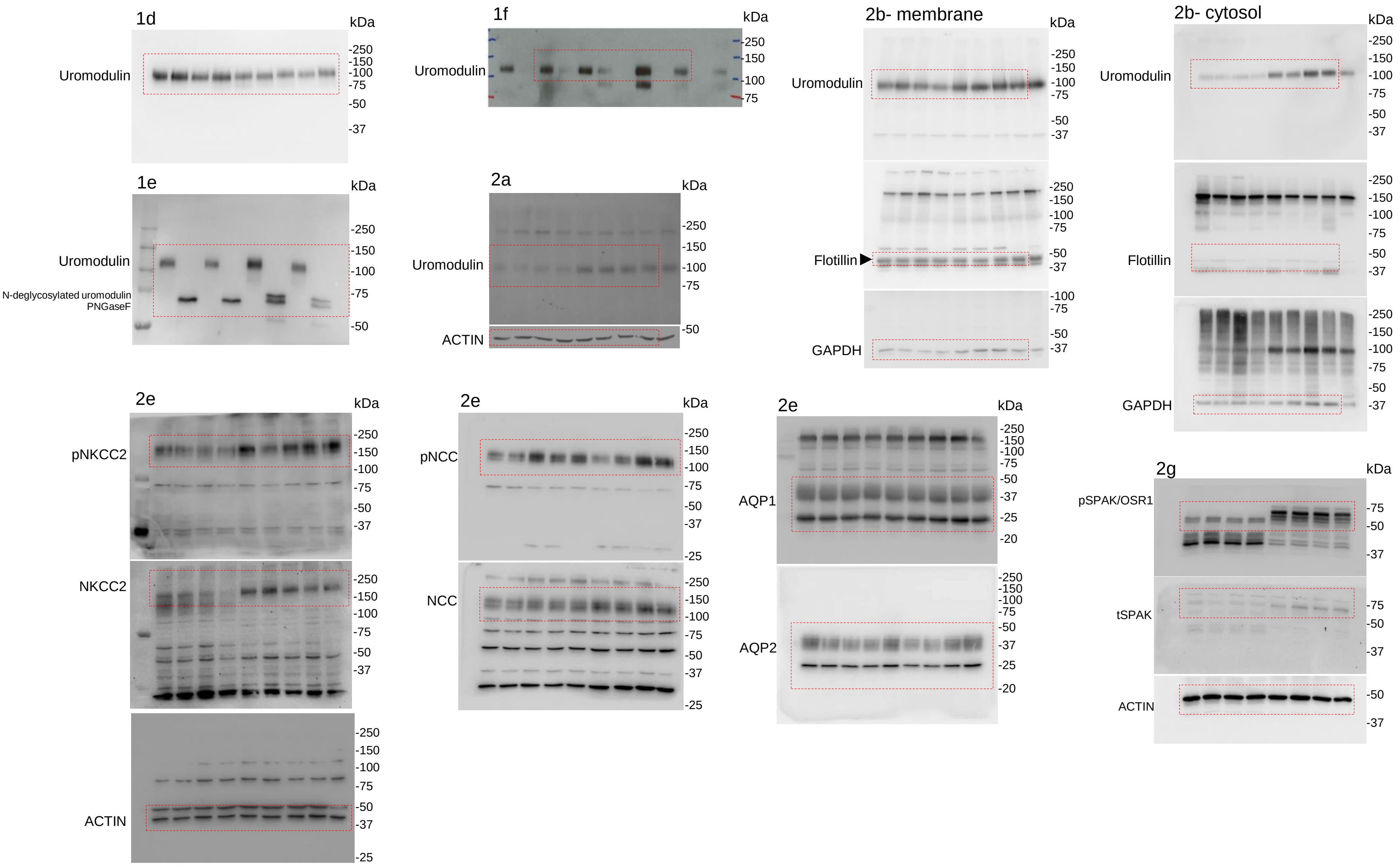

1d
kDa
Uromodulin
-250
-150
-100
-75
-50
-37
1f
kDa
Uromodulin
-250
-150
-100
-75
2b- membrane
kDa
Uromodulin
-250
-150
-100
-75
-50
-37
Flotillin
-250
-150
-100
-75
-50
-37
GAPDH
-100
-75
-50
-37
2b- cytosol
kDa
Uromodulin
-250
-150
-100
-75
-50
-37
Flotillin
-250
-150
-100
-75
-50
-37
GAPDH
-250
-150
-100
-75
-50
-37
1e
kDa
Uromodulin
N-deglycosylated uromodulin
PNGaseF
-250
-150
-100
-75
-50
2a
kDa
Uromodulin
ACTIN
-250
-150
-100
-75
-50
2e
kDa
pNKCC2
NKCC2
ACTIN
-250
-150
-100
-75
-50
-37
-25
2e
kDa
pNCC
NCC
-250
-150
-100
-75
-50
-37
-25
2e
kDa
AQP1
AQP2
-250
-150
-100
-75
-50
-37
-25
-20
2g
kDa
pSPAK/OSR1
tSPAK
ACTIN
-75
-50
-37

Suppl. Fig. S8 (continued)

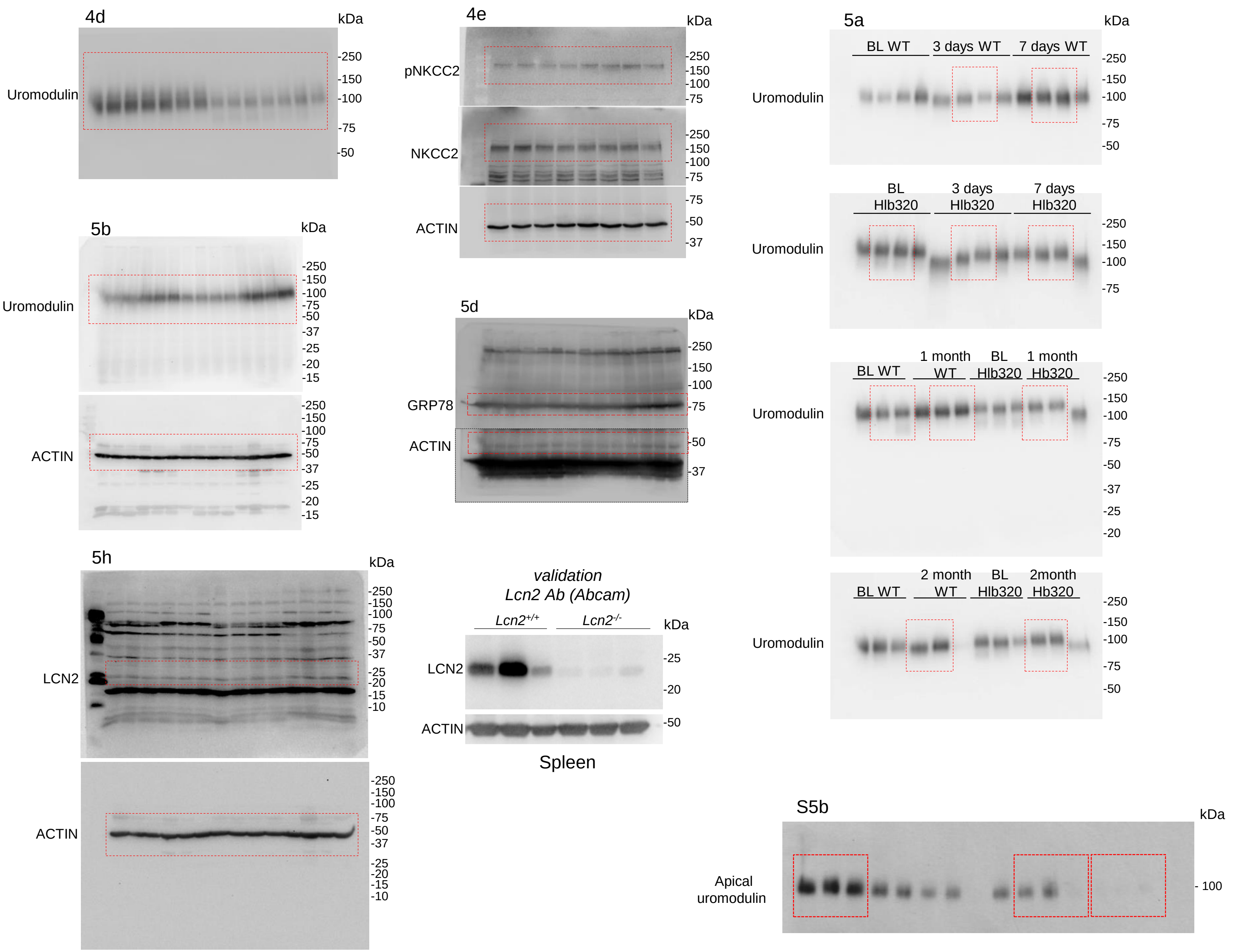

Suppl. Table S1: Primers used in real-time RT-PCR analyses.

Gene product	Forward primer (5'-3')	Reverse primer (5'-3')	PCR Product (bp)	Efficiency
18S	GTA ACC CGT TGA ACC CCA TT	CCA TCC AAT CGG TAG TAG CG	151	0.98±0.02
36B4	CTT CAT TGT GGG AGC AGA CA	TTC TCC AGA GCT GGG TTG TT	150	1.02±0.02
Actb	TGC CCA TCT ATG AGG GCT AC	CCC GTT CAG TCA GGA TCT TC	102	1.03 ± 0.04
Aqp1	GCT GTC ATG TAC ATC ATC GCC CAG	AGG TCA TTG CGG CCA AGT GAA T	107	1.02 ± 0.03
Aqp2	TCA CTG GGT CTT CTG GAT CG	CGT TCC TCC CAG TCA GTG T	147	1.03 ± 0.04
Dpp4	GAA ATG CCA GGA GGA AGA AA	CCC AAC ATC CCA GCT GAT AG	151	1.01 ± 0.03
Gapdh	TGC ACC ACC AAC TGC TTA GC	GGA TGC AGG GAT GGG GGA GA	176	1.04 ± 0.03
Hepsin	CTG ACT GCT GCA CAT TGC TT	GGG TCT CGA AAG GGA AGG TA	152	0.98 ± 0.02
Hprt 1	ACA TTG TGG CCC TCT GTG TG	TTA TGT CCC CCG TTG ACT GA	162	0.99 ± 0.01
Kcnj1	CCG TGT TCA TCA CAG CCT TCT T	CCG TAA CCT ATG GTC ACT TGG G	190	1.03 ± 0.03
Lcn2	ATG TCA CCT CCA TCC TGG TC	GTG GCC ACT TGC ACA TTG TA	148	0.99 ± 0.03
Lrp2	CAG TGG ATT GGG TAG CAG GA	GCT TGG GGT CAA CAA CGA TA	150	0.98 ± 0.03
Muc1	CGG AGA TTT TCT GGG GAT CT	TCA TCT GCC TCC TTC TTA TGC	141	1.02 ± 0.04
Ppia	CGT CTC CTT CGA GCT GTT TG	CCA CCC TGG CAC ATG AAT C	139	1.02 ± 0.02
Prss8	ATC ACC CAC TCA AGC TAC CG	AGT ACA GTG AAG GCC GTT GG	147	1.03 ± 0.03
Scnn1b	CCA ACC CTG GGA CTG AAT TT	GGC ATA GAT GCC CTC CTC T	149	1.02 ± 0.04
Scnn1g	GTG GCT GTG CCC AGT ACA G	CAG GAT TGC TTG CAC ACT GA	151	0.98 ± 0.04
Slc12a1a	TGG GTT GTC AAC TTC TGC AA	AGC AAA GAT CAA GCC TAT TGA CC	118	1.03 ± 0.03
Slc12a1b	ACA GGT TTG TCC ACC TCT GC	AGC AAA GAT CAA GCC TAT TGA CC	120	0.99 ± 0.04
Slc12a1f	ATT GGC CTG AGC GTA GTT GT	AGC AAA GAT CAA GCC TAT TGA CC	150	1.02 ± 0.04
Slc12a3	CAT GGT CTC CTT TGC CAA CT	TGC CAA AGA AGC TAC CAT CA	148	1.01 ± 0.03
Umod	TCA GCC TGA AGA CCT CCC TA	GAA AAG CCT CAG TGG ACA GC	156	0.98 ± 0.03

The primers were designed using Beacon Design 2.0 (Premier Biosoft International, Palo Alto, CA).

References

1. Brunati, M, Perucca, S, Han, L, Cattaneo, A, Consolato, F, Andolfo, A, et al.: The serine protease hepsin mediates urinary secretion and polymerisation of Zona Pellucida domain protein uromodulin. *Elife,* 4**:** e08887, 2015.
